# Molecular temperature descriptors as a novel approach for QSPR analysis of Borophene nanosheets

**DOI:** 10.1371/journal.pone.0302157

**Published:** 2024-06-18

**Authors:** Abdul Rauf Khan, Zafar Ullah, Muhammad Imran, Sidra Aziz Malik, Lamis M. Alamoudi, Murat Cancan

**Affiliations:** 1 Department of Mathematics, Faculty of Sciences, Ghazi University, Dera Ghazi Khan, Pakistan; 2 Division of Science, Department of Mathematics, University of Education, Lahore, Pakistan; 3 Department of Mathematical Sciences, United Arab Emirates University, Al Ain, United Arab Emirates; 4 Department of Statistics, King Abdulaziz University, Jeddah, Saudi Arabia; 5 Faculty of Education, Yuzuncu Yil University, Van, Turkey; Federal University of ABC, BRAZIL

## Abstract

Borophene nanosheets appear in various sizes and shapes, ranging from simple planar structures to complicated polyhedral formations. Due to their unique chemical, optical, and electrical properties, Borophene nanosheets are theoretically and practically attractive and because of their high thermal conductivity, boron nanosheets are suitable for efficient heat transmission applications. In this paper, temperature indices of borophene nanosheets are computed and these indices are employed in QSPR analysis of attributes like Young’s modulus, Shear modulus, and Poisson’s ratio of borophene nanosheets and borophene *β*_12_ sheets. The regression model for the F-Temperature index is discovered to be the best fit for shear modulus, the reciprocal product connectivity temperature index is discovered to be fit for Poisson’s ratio and the second hyper temperature index is discovered to be fit for Young’s modulus based on the correlation coefficient.

## 1 Introduction

Boron was simultaneously discovered in 1808 by DAVY in England and France by GAY-LUSSAC, and THENARD used separate methods [1]. A unique element called boron shares a periodic table element with its neighbour, carbon, which can form bonds formed by atoms from the same element. This characteristic enables the generation of various boron hydrides and the heteroatom variants of hydrides, collectively called Borophene nanosheets [2, 3]. As the name indicates, Borophene nanosheets have a polyhedral cage-like structure compared to organic compounds, which are made up of chains and rings. Atoms of boron or heteroatoms that comprise this structure are engaged on multiple edges, ranging from the highly brittle quadrilateral Borophene nanosheet to the compounds built out of the octahedral Borophene nanosheet [4, 5]. This stability is due to the delocalization of bonds of the 3-dimensional aromatic structure of Borophene nanosheet compounds, which differs from the 2D aromaticity of organic compounds [6]. The astonishing structural variety of Borophene nanosheets has recently been uncovered thanks to experimental methods combined with theoretical analysis [7]. Despite the well-established two to three-dimensional transition thresholds, the parameters that determine the geometry that these systems acquire are unknown. Doping frequently causes structural changes that result in the lowest energy structure having an utterly different boron skeleton than the cluster without doping [8]. Borophene nanosheets have remarkable properties in antimicrobial therapy, drug design [9], cancer therapy [10], pharmacophores [11], used as broadband membrane carriers [12], for the synthesis of liquid crystals [13] and luminescent materials [14]. The effectiveness of Borophene nanosheet derivatives against common and multi-drug resistant disease strains is identical. They also exhibit anti-biofilm activity and are less likely to result in medication resistance. Dendrimers, coordinated polymers, metal-organic systems, polymer compounds, and non-linear optical materials have all been made using Borophene nanosheets. These clusters have been employed in nanoscience as the molecules’ fundamental components [15].

In 2015, researchers from a variety of domains were drawn to Borophene, a 2D boron sheet that was successfully synthesized on Ag substrates. It is the lightest 2D material and has similar properties to graphene. Borophene is superconductive and has the highest pressure and external strain among 2D materials. To assess its suitability for use in a variety of disciplines, a great deal of research has been done on its stability, electrical qualities, and chemical and structural complexity [16]. Borophene’s mechanical properties are significant, including low mass density, high strength, and high in-plane stiffness, making it suitable for composite design and flexible nano device fabrication. Its anisotropic structure allows for effective control of magnetic and electronic properties making it a suitable candidate in various applications. Borophene’s polymorphic nature and strong electron-phonon coupling make it a valuable material for future applications due to its resources, low cost, and excellent electrical performance [17, 18]. Despite the wide range of possible uses for Borophene, one of the most difficult tasks that limit its application involves the synthetic methodology and identification of its neoatomic forms with carefully thought-out structure-property connections. Furthermore, in the case of synthetic 2D materials, a variety of parameters, including component elements, processing conditions, and growth substrates, impact the final atomic structure. The assured synthesis of quality specimens and the separation of Borophene from substrates remain difficult and need ongoing theoretical and experimental work to achieve practical applications [19, 20]. With its good metal properties, Borophene has potential in energy-related applications such as lithium batteries and biosensors. Its electrical sensitivities make it suitable for sequencing DNA and gas sensors. However, its NIR absorption needs improvement. Despite extensive research, applications are limited due to low yield and limited nanosheet size [21].

Researchers have characterized the properties of Borophene, a 2-dimensional material, to find its advantages over graphene and other materials. Borophene is a superconductor that is more robust, flexible, and an excellent heat and electrical conductor. Depending on how the voids are arranged and oriented, their properties can be adjusted. Its amazing mechanical capabilities and orientationally adjustable qualities make a lighter, electron-rich Borophene an equivalent contender to graphene. Borophene is also a superior substitute for graphene in composite progress since it has a greater Young’s modulus. Compared to other 2D materials, it is a superior conductor of heat and electricity due to its remarkable anisotropic behaviour [22–24].

Kulli initiated in 2020 a graph’s temperature indices [25]. In chemical graph theory, these indices are used to quantify the chemical characteristics of chemical compounds. These indies are computed for a molecular graph silicate network and a silicate chain network and present valuable results in [26, 27]. Various graph-theoretic techniques and algorithms can be used to analyze the structure and behaviour of the chemical network once it has been converted into a graph [28]. This approach can be used to discover novel chemical reactions or processes and to understand the operation of complex chemical systems [29]. Chemical networks, physical, chemical, and thermal properties, as well as biological and chemical activities, can all be evaluated using chemical graph theory [30]. It is possible to determine a molecule’s chemical, physical, and biological properties using its topological index [31]. Graph theory is extensively employed in modern chemistry, mainly organic chemistry. Mathematical chemistry has demonstrated that polynomials and functions can uncover instructions concealed in the symmetry of chemical graphs. Topological indices can be used to evaluate QSPR, QSAR, and other chemical applications based on theory [32]. Diverse fields of study have investigated topological indices to fathom various types of graphs. In quantitative structure-activity and structure-property interactions, topological indices are employed as numerical descriptions to compare molecules’ biological, physical, and chemical aspects. Numerous researchers have examined various chemical compounds in recent years and computed topological descriptors of different molecular graphs.

Graph indices can be used for various purposes, including chemical record keeping, establishing relationships between structure and activity, property and relationship, and property and relationship [33]. Mathematicians have developed novel concepts with the use of graph indices [34].

We exclusively consider limited, simple, and connected graphs in this work. Assume that *K* is a graph, where *E*_*K*_ represents the set of edges, and *V*_*K*_ represents the set of vertices. The degree is determined by the number of vertices surrounding a point *u*. We advise the individual to search for simple terms and symbols. Fajtlowicz presented the temperature of a graph [35]:

$$\begin{array}{c} T_{\psi_{i}}=\frac{d_{\psi_{i}}}{|V_{K}|-d_{\psi_{i}}}\;\;\;where\;\;\;\forall \;\;\psi_{i}\in V_{K} \end{array}$$ (1)

The first temperature index is defined as below [36]:

$$\begin{array}{c} T_{1}\left(K\right)=\sum_{\psi \rho \in E(K)}(T_{\psi }+T_{\rho }) \end{array}$$ (2)

The second temperature index was introduced by Kulli in 2020 in [37],

$$\begin{array}{c} T_{2}\left(K\right)=\sum_{\psi \rho \in E(K)}(T_{\psi }\times T_{\rho }) \end{array}$$ (3)

Kulli defined the first and second hyper temperature indices in [37],

$$\begin{array}{c} {\text{HT}}_{1}\left(K\right)=\sum_{\psi \rho \in E(K)}{\left(T_{\psi }+T_{\rho }\right)}^{2} \end{array}$$ (4)

$$\begin{array}{c} {\text{HT}}_{2}\left(K\right)=\sum_{\psi \rho \in E(K)}{\left(T_{\psi }\times T_{\rho }\right)}^{2} \end{array}$$ (5)

The same study provides more related topological indices [37]. The reciprocal product connectivity index, the product connectivity temperature index, and the sum connectivity temperature index were given, respectively as

$$\begin{array}{c} \text{RPT}\left(K\right)=\sum_{\psi \rho \in E(K)}\sqrt{\left(T_{\psi }\times T_{\rho }\right)} \end{array}$$ (6)

$$\begin{array}{c} \text{PT}\left(K\right)=\sum_{\psi \rho \in E(K)}\frac{1}{\sqrt{\left(T_{\psi }\times T_{\rho }\right)}} \end{array}$$ (7)

$$\begin{array}{c} \text{ST}\left(K\right)=\sum_{\psi \rho \in E(K)}\frac{1}{\sqrt{\left(T_{\psi }+T_{\rho }\right)}} \end{array}$$ (8)

Kulli also examined the F-temperature index of a graph *K* in [37], as given below

$$\begin{array}{c} \text{FT}\left(K\right)=\sum_{\psi \rho \in E(K)}(T_{\psi }^{2}+T_{\rho }^{2}) \end{array}$$ (9)

In this article, the above-defined eight temperature indices are constructed by the atom-bonds partition of Borophene nanosheet $BN_{\left(l,m\right)}$.

## 2 Borophene nanosheet $BN_{\left(l,m\right)}$

**Fig 1:**
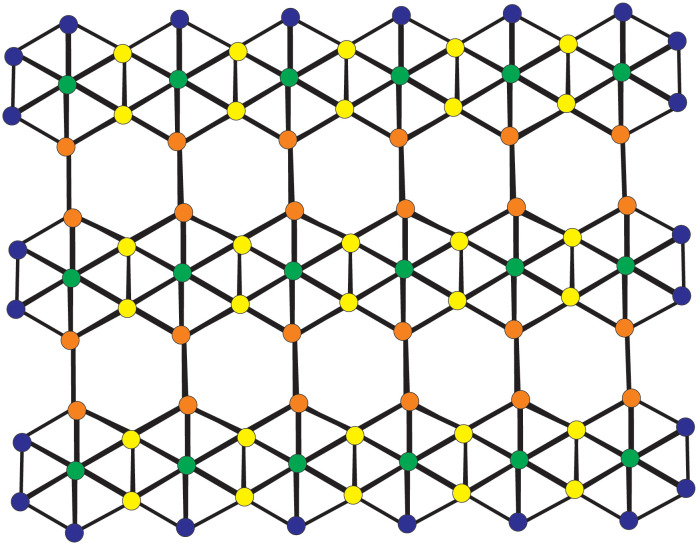
Borophene nanosheets for l = 6 and m = 3.

Borophene nanosheets are three-dimensional formations comprised of boron atoms that are small in size. They appear in various sizes and shapes, ranging from simple planar structures to complicated polyhedral formations. Due to their unique chemical, optical, and electrical properties, Borophene nanosheets are theoretically and practically attractive. Two-dimensional Borophene nanosheet, composed of Borophene nanosheets, has recently received greater interest [38]. Due to their high thermal conductivity, boron nanosheets are suitable for efficient heat transmission applications. Due to their triangular arrangement of the boron atoms, strong covalent bonding occurs, providing the sheet tremendous strength and stiffness. Borophene nanosheets have unique electronic properties because of their 2D structure and boron atoms’ electrical configuration, making them useful in electronic and optoelectronic devices. Because of the sheet’s closely bound boron atoms, it is relatively secure and impenetrable to chemical reactions [39]. Various industries, like electronics, optoelectronics, and energy storage, may utilize boron for their products [40–42]. The Borophene nanosheet, borophene’s physical and chemical characteristics, and its application are given in [43–50]. Borophene nanosheet *γ*-sheet for the *l* = 6, *m* = 3 is sketched in Fig 1 and for *l* = 2, *m* = 3 is drawn in Fig 1. Here, in Borophene nanosheet $BN_{\left(l,m\right)}$, there are nine types of atom-bonds based on the valency of every atom of $BN_{\left(l,m\right)}$, which are (3 ∼ 3), (3 ∼ 4), (3 ∼ 5), (3 ∼ 6), (4 ∼ 4), (4 ∼ 5), (4 ∼ 6), (5 ∼ 5) and (5 ∼ 6) in $BN_{\left(l,m\right)}$. Tables 1 and 2 represent atom bond partitions based on valency. The atom-bonds partition of $BN_{\left(l,m\right)}$ given as: The sum of all the atoms and the bonds of $BN_{\left(l,m\right)}$:

$$\begin{array}{c} |V(BN_{\left(l,m\right)}|=5lm+2m\;\;and\;\;|E(BN_{\left(l,m\right)}|=12lm+m-l \end{array}$$

Using edge partition given in Tables 1 and 2 along with Eq (1), we get Tables 3–5 for $BN_{\left(l,m\right)}$.

**Table 1:** Edge partition of Borophene nanosheet $BN_{\left(l,m\right)}$.

Edge Partition	*E*_(3,3)_	*E*_(3,4)_	*E*_(3,5)_	*E*_(3,6)_	*E*_(4,4)_
Cardinality	2m+4	2m-4	4l -4	2l+4m	l(m-1)

**Table 2:** Edge partition of Borophene nanosheet $BN_{\left(l,m\right)}$.

Edge Partition	*E*_(4,5)_	*E*_(4,6)_	*E*_(5,5)_	*E*_(5,6)_
Cardinality	4(l-1)(m-1)	2l(m-1)	m(l-1)	4m(l-1)

**Table 3:** Atomic-bond partition of $BN_{\left(l,m\right)}$.

(*T*_*u*_, *T*_*v*_)	$\left(\frac{3}{\left(5lm+2m-3\right)},\frac{3}{\left(5lm+2m-3\right)}\right)$	$\left(\frac{3}{\left(5lm+2m-3\right)},\frac{4}{\left(5lm+2m-4\right)}\right)$	$\left(\frac{3}{\left(5lm+2m-3\right)},\frac{5}{\left(5lm+2m-5\right)}\right)$
Frequency	2*m* + 4	4*m* − 4	4*l* − 4

**Table 4:** Atomic-bond partition of $BN_{\left(l,m)\right)}$.

(*T*_*u*_, *T*_*v*_)	$\left(\frac{3}{\left(5lm+2m-3\right)},\frac{6}{\left(5lm+2m-6\right)}\right)$	$\left(\frac{4}{\left(5lm+2m-4\right)},\frac{4}{\left(5lm+2m-4\right)}\right)$	$\left(\frac{4}{\left(5lm+2m-4\right)},\frac{5}{\left(5lm+2m-5\right)}\right)$
Frequency	2*l* + 4*m*	*l*(*m* − 1)	4(*l* − 1)(*m* − 1)

**Table 5:** Atomic-bond partition of $BN_{\left(l,m)\right)}$.

(*T*_*u*_, *T*_*v*_)	$\left(\frac{4}{\left(5lm+2m-4\right)},\frac{6}{\left(5lm+2m-6\right)}\right)$	$\left(\frac{5}{\left(5lm+2m-5\right)},\frac{5}{\left(5lm+2m-5\right)}\right)$	$\left(\frac{5}{\left(5lm+2m-5\right)},\frac{6}{\left(5lm+2m-6\right)}\right)$
Frequency	2*l*(*m* − 1)	(*l* − 1)*m*	4*m*(*l* − 1)

### 2.1 Temperature indices for Borophene nanosheet $BN_{\left(l,m\right)}$

In this section, we present some computed results.

1. Let $BN_{\left(l,m\right)}$ be a Borophene nanosheet. Then the first temperature index of $BN_{\left(l,m\right)}$ is $\frac{6(2m+4)}{5lm+2m-3}+\frac{\left(4m-4)(35lm+14m-24\right)}{\left(5lm+2m-3)(5lm+2m-4\right)}+\frac{\left(4l-4)(40lm+16m-30\right)}{\left(5lm+2m-3)(5lm+2m-5\right)}+\frac{\left(2l+4m)(45lm+18m-36\right)}{\left(5lm+2m-3)(5lm+2m-6\right)}+\frac{8(lm-l)}{\left(5lm+2m-4\right)}+\frac{\left(4lm-4l-4m+4)(45lm+18m-40\right)}{\left(5lm+2m-4)(5lm+2m-5\right)}+\frac{\left(2lm-l)(50lm+20m-48\right)}{\left(5lm+2m-4)(5lm+2m-6\right)}+\frac{10(lm-m)}{\left(5lm+2m-5\right)}+\frac{\left(4lm-4)(55lm+22m-60\right)}{\left(5lm+2m-5)(5lm+2m-6\right)}$.

Tables 3–5 along with Eq (2), implies

$$\begin{array}{ccc} T_{1}\left(BN_{\left(l,m\right)}\right) & = & \left(2m+4\right)\left[\frac{3}{(5lm+2m)-3}+\frac{3}{(5lm+2m)-3}\right]\\ & + & \left(4m-4\right)[\frac{3}{(5lm+2m)-3}+\frac{4}{(5lm+2m)-4}]\\ & + & \left(4l-4\right)[\frac{3}{(5lm+2m)-3}+\frac{5}{(5lm+2m)-5}]\\ & + & \left(2l+4m\right)[\frac{3}{(5lm+2m)-3}+\frac{6}{(5lm+2m)-6}]\\ & + & \left(lm-l\right)[\frac{4}{(5lm+2m)-4}+\frac{4}{(5lm+2m)-4}]\\ & + & \left(4lm-4l-4m+4\right)[\frac{4}{(5lm+2m)-4}+\frac{5}{(5lm+2m)-5}]\\ & + & \left(2lm-2l\right)[\frac{4}{(5lm+2m)-4}+\frac{6}{(5lm+2m)-6}]\\ & + & \left(lm-m\right)[\frac{5}{(5lm+2m)-5}+\frac{5}{(5lm+2m)-5}]\\ & + & \left(4lm-4m\right)[\frac{5}{(5lm+2m)-5}+\frac{6}{(5lm+2m)-6}] \end{array}$$

After simplification, we get

$$\begin{array}{ccc} T_{1}\left(BN_{\left(l,m\right)}\right) & = & \frac{6(2m+4)}{(5lm+2m)-3}+\frac{\left(4m-4)(35lm+14m-24\right)}{\left(5lm+2m-3)(5lm+2m-4\right)}\\ & + & \frac{\left(4l-4)(40lm+16m-30\right)}{\left(5lm+2m-3)(5lm+2m-5\right)}+\frac{\left(2l+4m)(45lm+18m-36\right)}{\left(5lm+2m-3)(5lm+2m-6\right)}\\ & + & \frac{8(lm-l)}{\left(5lm+2m-4\right)}+\frac{\left(4lm-4l-4m+4)(45lm+18m-40\right)}{\left(5lm+2m-4)(5lm+2m-5\right)}\\ & + & \frac{\left(2lm-l)(50lm+20m-48\right)}{\left(5lm+2m-4)(5lm+2m-6\right)}+\frac{10(lm-m)}{\left(5lm+2m-5\right)}\\ & + & \frac{\left(4lm-4m)(55lm+22m-60\right)}{\left(5lm+2m-5)(5lm+2m-6\right)}. \end{array}$$

2. Let $BN_{\left(l,m\right)}$ be a Borophene nanosheet. Then the second temperature index is $\frac{9(2m+4)}{{\left(5lm+2m-3\right)}^{2}}+\frac{12(4m-4)}{\left(5lm+2m-3)(5lm+2m-4\right)}+\frac{15(4l-4)}{\left(5lm+2m-3)(5lm+2m-5\right)}+\frac{18(2l+4m)}{\left(5lm+2m-3)(5lm+2m-6\right)}+\frac{16(lm-l)}{{\left(5lm+2m-4\right)}^{2}}+\frac{20(4lm-4l-4m+4)}{\left(5lm+2m-4)(5lm+2m-5\right)}+\frac{24(2lm-2l)}{\left(5lm+2m-4)(5lm+2m-6\right)}+\frac{25(lm-m)}{{\left(5lm+2m-5\right)}^{2}}+\frac{30(4lm-4m)}{\left(5lm+2m-5)(5lm+2m-6\right)}$.

Tables 3–5 along with Eq (3), gives

$$\begin{array}{ccc} T_{2}\left(BN_{\left(l,m\right)}\right) & = & \left(2m+4\right)[\frac{3}{(5lm+2m)-3}\times \frac{3}{(5lm+2m)-3}]\\ & + & \left(4m-4\right)[\frac{3}{(5lm+2m)-3}\times \frac{4}{(5lm+2m)-4}]\\ & + & \left(4l-4\right)[\frac{3}{(5lm+2m)-3}\times \frac{5}{(5lm+2m)-5}]\\ & + & \left(2l+4m\right)[\frac{3}{(5lm+2m)-3}\times \frac{6}{(5lm+2m)-6}]\\ & + & \left(lm-l\right)[\frac{4}{(5lm+2m)-4}\times \frac{4}{(5lm+2m)-4}]\\ & + & \left(4lm-4l-4m+4\right)[\frac{4}{(5lm+2m)-4}\times \frac{5}{(5lm+2m)-5}]\\ & + & \left(2lm-2l\right)[\frac{4}{(5lm+2m)-4}\times \frac{6}{(5lm+2m)-6}]\\ & + & \left(lm-m\right)[\frac{5}{(5lm+2m)-5}\times \frac{5}{(5lm+2m)-5}]\\ & + & \left(4lm-4m\right)[\frac{5}{(5lm+2m)-5}\times \frac{6}{(5lm+2m)-6}] \end{array}$$

After simplification, we get

$$\begin{array}{ccc} T_{2}\left(BN_{\left(l,m\right)}\right) & = & \frac{9(2m+4)}{{\left(5lm+2m-3\right)}^{2}}+\frac{12(4m-4)}{\left(5lm+2m-3)(5lm+2l-4\right)}\\ & + & \frac{15(4l-4)}{\left(5lm+2m-3)(5lm+2m-5\right)}\\ & + & \frac{18(2l+4m)}{\left(5lm+2m-3)(5lm+2l-6\right)}+\frac{16(lm-l)}{{\left(5lm+2m-4\right)}^{2}}\\ & + & \frac{20(4lm-4l-4m+4)}{\left(5lm+2m-4)(5lm+2l-5\right)}\\ & + & \frac{24(2lm-2l)}{\left(5lm+2m-4)(5lm+2l-6\right)}+\frac{25(lm-m)}{{\left(5lm+2m-5\right)}^{2}}\\ & + & \frac{30(4lm-4m)}{\left(5lm+2m-5)(5lm+2l-6\right)}. \end{array}$$ (10)

3. Let $BN_{\left(l,m\right)}$ be Borophene nanosheet. Then the first hyper temperature index is $\frac{36(2m+4)}{{\left(5lm+2m-3\right)}^{2}}+\frac{\left(4m-4\right){\left(35lm+14m-24\right)}^{2}}{{\left(5lm+2m-3\right)}^{2}{\left(5lm+2m-4\right)}^{2}}+\frac{4\left(4l-4\right){\left(20lm+8m-15\right)}^{2}}{{\left(5lm+2m-3\right)}^{2}{\left(5lm+2m-5\right)}^{2}}+\frac{81\left(2l+4m\right){\left(5lm+2m-4\right)}^{2}}{{\left(5lm+2m-3\right)}^{2}{\left(5lm+2m-6\right)}^{2}}+\frac{64(lm-l)}{{\left(5lm+2m-4\right)}^{2}}+\frac{\left(4lm-4l-4m+4\right){\left(45lm+18m-40\right)}^{2}}{{\left(5lm+2m-4\right)}^{2}{\left(5lm+2m-5\right)}^{2}}+\frac{4\left(2lm-2l\right){\left(25lm+10m-24\right)}^{2}}{{\left(5lm+2m-4\right)}^{2}{\left(5lm+2m-6\right)}^{2}}+\frac{100(lm-m)}{{\left(5lm+2m-5\right)}^{2}}+\frac{\left(4lm-4m\right){\left(55lm+2m-60\right)}^{2}}{{\left(5lm+2m-5\right)}^{2}{\left(5lm+2m-6\right)}^{2}}$.

From Tables 3–5 along with Eq (4), we obtain

$$\begin{array}{ccc} HT_{1}\left(BN_{\left(l,m\right)}\right) & = & \left(2m+4\right){\left[\frac{3}{(5lm+2m)-3}+\frac{3}{(5lm+2m)-3}\right]}^{2}\\ & + & \left(4m-4\right){\left[\frac{3}{(5lm+2m)-3}+\frac{4}{(5lm+2m)-4}\right]}^{2}\\ & + & \left(4l-4\right){\left[\frac{3}{(5lm+2m)-3}+\frac{5}{(5lm+2m)-5}\right]}^{2}\\ & + & \left(2l+4m\right){\left[\frac{3}{(5lm+2m)-3}+\frac{6}{(5lm+2m)-6}\right]}^{2}\\ & + & \left(lm-l\right){\left[\frac{4}{(5lm+2m)-4}+\frac{4}{(5lm+2m)-4}\right]}^{2}\\ & + & \left(4lm-4l-4m+4\right){\left[\frac{4}{(5lm+2m)-4}+\frac{5}{(5lm+2m)-5}\right]}^{2}\\ & + & \left(2lm-2l\right){\left[\frac{4}{(5lm+2m)-4}+\frac{6}{(5lm+2m)-6}\right]}^{2}\\ & + & \left(lm-m\right){\left[\frac{5}{(5lm+2m)-5}+\frac{5}{(5lm+2m)-5}\right]}^{2}\\ & + & \left(4lm-4m\right){\left[\frac{5}{(5lm+2m)-5}+\frac{6}{(5lm+2m)-6}\right]}^{2} \end{array}$$

After simplification, we get

$$\begin{array}{ccc} HT_{1}\left(B{\text{CL}}_{l,m)}\right) & = & \frac{36(2m+4)}{{\left(5lm+2m-3\right)}^{2}}+\frac{\left(4m-4\right){\left(35lm+14m-24\right)}^{2}}{{\left(5lm+2m-3\right)}^{2}{\left(5lm+2m-4\right)}^{2}}\\ & + & \frac{4(4l-4)(20lm+8m-15)}{{\left(5lm+2m-3\right)}^{(}{5lm+2m-5)}^{2}}\\ & + & \frac{9\left(2l+4m\right){\left(15lm+6m-12\right)}^{2}}{{\left(5lm+2m-3\right)}^{2}{\left(5lm+2m-6\right)}^{2}}\\ & + & \frac{64(lm-l)}{{\left(5lm+2m-4\right)}^{2}}+\frac{\left(4lm-4l-4m+4\right){\left(45lm+18m-40\right)}^{2}}{{\left(5lm+2m-4\right)}^{2}{\left(5lm+2m-5\right)}^{2}}\\ & + & \frac{4\left(2lm-2l\right){\left(25lm+10m-24\right)}^{2}}{{\left(5lm+2m-4\right)}^{2}{\left(5lm+2m-6\right)}^{2}}+\frac{100(lm-m)}{{\left(5lm+2m-5\right)}^{2}}\\ & + & \frac{\left(4lm-4m\right){\left(55lm+2m-60\right)}^{2}}{{\left(5lm+2m-5\right)}^{2}{\left(5lm+2m-6\right)}^{2}}. \end{array}$$

4. Let $BN_{\left(l,m\right)}$ be Borophene nanosheet. Then the second hyper temperature index is $\frac{81(2m+4)}{{\left(5lm+2m-3\right)}^{4}}+\frac{144(4m-4)}{{\left(5lm+2m-3\right)}^{2}{\left(5lm+2m-4\right)}^{2}}+\frac{225(4l-4)}{{\left(5lm+2m-3\right)}^{2}{\left(5lm+2m-5\right)}^{2}}+\frac{324(2l+4m)}{{\left(5lm+2m-3\right)}^{2}{\left(5lm+2m-6\right)}^{2}}+\frac{256(lm-l)}{{\left(5lm+2m-4\right)}^{4}}+\frac{400(4lm-4l-4m+4)}{{\left(5lm+2m-4\right)}^{2}{\left(5lm+2m-5\right)}^{2}}+\frac{576(2lm-2l)}{{\left(5lm+2m-4\right)}^{2}{\left(5lm+2m-6\right)}^{2}}+\frac{625(lm-m)}{{\left(5lm+2m-5\right)}^{4}}+\frac{900(4ml-4m)}{{\left(5lm+2m-5\right)}^{2}{\left(5lm+2m-6\right)}^{2}}.$

From Tables 3–5 along with Eq (5), we attain

$$\begin{array}{ccc} HT_{2}\left(BN_{\left(l,m\right)}\right) & = & \left(2m+4\right){\left[\frac{3}{(5lm+2m)-3}\times \frac{3}{(5lm+2m)-3}\right]}^{2}\\ & + & \left(4m-4\right){\left[\frac{3}{(5lm+2m)-3}\times \frac{4}{(5lm+2m)-4}\right]}^{2}\\ & + & \left(4l-4\right){\left[\frac{3}{(5lm+2m)-3}\times \frac{5}{(5lm+2m)-5}\right]}^{2}\\ & + & \left(2l+4m\right){\left[\frac{3}{(5lm+2m)-3}\times \frac{6}{(5lm+2m)-6}\right]}^{2}\\ & + & \left(lm-l\right){\left[\frac{4}{(5lm+2m)-4}\times \frac{4}{(5lm+2m)-4}\right]}^{2}\\ & + & \left(4lm-4l-4m+4\right){\left[\frac{4}{(5lm+2m)-4}\times \frac{5}{(2lm+2m)-5}\right]}^{2}\\ & + & \left(2lm-2l\right){\left[\frac{4}{(5lm+2m)-4}\times \frac{5}{(5lm+2m)-5}\right]}^{2}\\ & + & \left(lm-m\right){\left[\frac{5}{(5lm+2m)-5}\times \frac{5}{(5lm+2m)-5}\right]}^{2}\\ & + & \left(4lm-4m\right){\left[\frac{5}{(5lm+2m)-5}\times \frac{6}{(2lm+2m)-6}\right]}^{2} \end{array}$$

After simplification, we get

$$\begin{array}{ccc} HT_{2}\left(BN_{\left(l,m\right)}\right) & = & \frac{81(2m+4)}{{\left(5lm+2m-3\right)}^{4}}+\frac{144(4m-4)}{{\left(5lm+2m-3\right)}^{2}{\left(5lm+2m-4\right)}^{2}}\\ & + & \frac{225(4l-4)}{{\left(5lm+2m-3\right)}^{2}{\left(5lm+2m-5\right)}^{2}}\\ & + & \frac{324(2l+4m)}{{\left(5lm+2m-3\right)}^{2}{\left(5lm+2m-6\right)}^{2}}\\ & + & \frac{256(lm-l)}{{\left(5lm+2m-4\right)}^{4}}+\frac{400(4lm-4l-4m+4)}{{\left(5lm+2m-4\right)}^{2}{\left(5lm+2m-5\right)}^{2}}\\ & + & \frac{576(2lm-2l)}{{\left(5lm+2m-4\right)}^{2}{\left(5lm+2m-6\right)}^{2}}+\frac{625(lm-m)}{{\left(5lm+2m-5\right)}^{4}}\\ & + & \frac{900(4lm-4m)}{{\left(5lm+2m-5\right)}^{2}{\left(5lm+2m-6\right)}^{2}}. \end{array}$$

5. Let $BN_{\left(l,m\right)}$ be Borophene nanosheet. Then the sum connectivity temperature index is $\frac{\left(2m+4\right)\sqrt{\left(5lm+2m-3\right)}}{\sqrt{6}}+\frac{\left(4m-4\right)\sqrt{\left(5lm+2m-3)(5lm+2m-4\right)}}{\sqrt{\left(35lm+14m-24\right)}}+\frac{\left(4l-4\right)\sqrt{\left(5lm+2m-3)(5lm+2m-5\right)}}{\sqrt{40lm+16m-30}}+\frac{\left(2l+4m\right)\sqrt{\left(5lm+2m-3)(5lm+2m-6\right)}}{\sqrt{45lm+18m-36}}+\frac{\left(lm-l\right)\sqrt{\left(5lm+2m-4\right)}}{\sqrt{8}}+\frac{\left(4lm-4l-4m+4\right)\sqrt{\left(5lm+2m-4)(5lm+2m-5\right)}}{\sqrt{45lm+18m-40}}+\frac{\left(2lm-2l\right)\sqrt{\left(5lm+2m-4)(5lm+2m-6\right)}}{\sqrt{50lm+20m-48}}+\frac{\left(lm-m\right)\sqrt{\left(5lm+2m-5\right)}}{\sqrt{10}}+\frac{\left(4lm-4m\right)\sqrt{\left(5lm+2m-5)(5lm+2m-6\right)}}{\sqrt{55lm+22m-60}}.$

From Tables 3–5 along with Eq (8), we get

$$\begin{array}{ccc} ST(BN_{\left(l,m\right)}) & = & \left(2m+4\right)\frac{1}{\sqrt{\left[\frac{3}{(5lm+2m)-3}+\frac{3}{(5lm+2m)-3}\right]}}\\ & + & \left(4m-4\right)\frac{1}{\sqrt{\left[\frac{3}{(5lm+2m)-3}+\frac{4}{(5lm+2m)-4}\right]}}\\ & + & \left(4l-4\right)\frac{1}{\sqrt{\left[\frac{3}{(5lm+2m)-3}+\frac{5}{(5lm+2m)-5}\right]}}\\ & + & \left(2l+4m\right)\frac{1}{\sqrt{\left[\frac{3}{(5lm+2m)-3}+\frac{6}{(5lm+2m)-6}\right]}}\\ & + & \left(lm-l\right)\frac{1}{\sqrt{\left[\frac{4}{(5lm+2m)-4}+\frac{4}{(5lm+2m)-4}\right]}}\\ & + & \left(4lm-4l-4m+4\right)\frac{1}{\sqrt{\left[\frac{4}{(5lm+2m)-4}+\frac{5}{(5lm+2m)-5}\right]}}\\ & + & \left(2lm-2l\right)\frac{1}{\sqrt{\left[\frac{4}{(5lm+2m)-4}+\frac{6}{(5lm+2m)-6}\right]}}\\ & + & \left(lm-m\right)\frac{1}{\sqrt{\left[\frac{5}{(5lm+2m)-5}+\frac{5}{(5lm+2m)-5}\right]}}\\ & + & \left(4lm-4m\right)\frac{1}{\sqrt{\left[\frac{5}{(5lm+2m)-5}+\frac{6}{(5lm+2m)-6}\right]}} \end{array}$$

After simplification, we get

$$\begin{array}{ccc} ST(BN_{\left(l,m\right)}) & = & \frac{\left(2m+4\right)\sqrt{\left(5lm+2m-3\right)}}{\sqrt{6}}\\ & + & \frac{\left(4m-4\right)\sqrt{\left(5lm+2m-3)(5lm+2m-4\right)}}{\sqrt{\left(35lm+14m-24\right)}}\\ & + & \frac{\left(4l-4\right)\sqrt{\left(5lm+2m-3)(5lm+2m-5\right)}}{\sqrt{40lm+16m-30}}\\ & + & \frac{\left(2l+4m\right)\sqrt{\left(5lm+2m-3)(5lm+2m-6\right)}}{\sqrt{45lm+18m-36}}\\ & + & \frac{\left(lm-l\right)\sqrt{\left(5lm+2m-4\right)}}{\sqrt{8}}\\ & + & \frac{\left(4lm-4l-4m+4\right)\sqrt{(5lm+2m-4)(5lm+2m-5}}{\sqrt{45lm+18m-40}}\\ & + & \frac{\left(2lm-2l\right)\sqrt{\left(5lm+2m-4)(5lm+2m-6\right)}}{\sqrt{50lm+20m-48}}\\ & + & \frac{\left(lm-m\right)\sqrt{\left(5lm+2m-5\right)}}{\sqrt{10}}\\ & + & \frac{\left(4lm-4m\right)\sqrt{\left(5lm+2m-5)(5lm+2m-6\right)}}{\sqrt{55lm+22m-60}}. \end{array}$$

6. Let $BN_{\left(l,m\right)}$ be Borophene nanosheet. Then the product connectivity temperature index is $\frac{10lm^{2}+4m^{2}+20lm+2m-12}{3}+\frac{\left(4m-4\right)\sqrt{\left(5lm+2m-3)(5lm+2m-4\right)}}{2\sqrt{3}}+\frac{\left(4l-4\right)\sqrt{\left(5lm+2m-3)(5lm+2m-5\right)}}{\sqrt{15}}+\frac{\left(2l+4m\right)\sqrt{\left(5lm+2m-3)(5lm+2m-6\right)}}{\sqrt{18}}+\frac{\left(2lm^{2}-6lm-4l\right)}{4}+\frac{\left(4lm-4l-4m+4\right)\sqrt{\left(5lm+2m-4)(5lm+2m-5\right)}}{2\sqrt{5}}+\frac{\left(2lm-2l\right)\sqrt{\left(5lm+2m-4)(5lm+2m-6\right)}}{2\sqrt{6}}+\frac{\left(5l^{2}m^{2}+2lm^{2}-10lm-2m+5\right)}{5}+\frac{\left(4lm-4m\right)\sqrt{\left(5lm+2m-5)(5lm+2m-6\right)}}{\sqrt{30}}.$

From Tables 3–5 along with Eq (7), we obtain

$$\begin{array}{ccc} PT(BN_{\left(l,m\right)}) & = & \left(2m+4\right)\frac{1}{\sqrt{\left[\frac{3}{(5lm+2m)-3}\times \frac{3}{(5lm+2m)-3}\right]}}\\ & + & \left(4m-4\right)\frac{1}{\sqrt{\left[\frac{3}{(5lm+2m)-3}\times \frac{4}{(5lm+2m)-4}\right]}}\\ & + & \left(4l-4\right)\frac{1}{\sqrt{\left[\frac{3}{(5lm+2m-3}\times \frac{5}{\left(5lm+2m-5\right)}\right]}}\\ & + & \left(2l+4m\right)\frac{1}{\sqrt{\left[\frac{3}{(5lm+2m)-3}\times \frac{6}{(5lm+2m)-6}\right]}}\\ & + & \left(lm-l\right)\frac{1}{\sqrt{\left[\frac{4}{(5lm+2m)-4}\times \frac{4}{(5lm+2m)-4}\right]}}\\ & + & \left(4lm-4l-4m+4\right)\frac{1}{\sqrt{\left[\frac{4}{(5lm+2m)-4}\times \frac{5}{(5lm+2m)-5}\right]}}\\ & + & \left(2lm-2l\right)\frac{1}{\sqrt{\left[\frac{4}{(5lm+2m)-4}\times \frac{6}{(5lm+2m)-6}\right]}}\\ & + & \left(lm-1\right)\frac{1}{\sqrt{\left[\frac{5}{(5lm+2m)-5}\times \frac{5}{(5lm+2m)-5}\right]}}\\ & + & \left(4lm-4m\right)\frac{1}{\sqrt{\left[\frac{5}{(5lm+2m)-5}\times \frac{6}{(5lm+2m)-6}\right]}} \end{array}$$

After simplification, we get

$$\begin{array}{ccc} PT(BN_{\left(l,m\right)}) & = & \frac{10lm^{2}+4m^{2}+20lm+2m-12}{3}\\ & + & \frac{\left(4m-4\right)\sqrt{\left(5lm+2m-3)(5lm+2m-4\right)}}{2\sqrt{3}}\\ & + & \frac{\left(4l-4\right)\sqrt{\left(5lm+2m-3)(5lm+2m-5\right)}}{\sqrt{15}}\\ & + & \frac{\left(2l+4m\right)\sqrt{\left(5lm+2m-3)(5lm+2m-6\right)}}{\sqrt{18}}+\frac{\left(2lm^{2}-6lm-4l\right)}{4}\\ & + & \frac{\left(4lm-4l-4m+4\right)\sqrt{\left(5lm+2m-4)(5lm+2m-5\right)}}{2\sqrt{5}}\\ & + & \frac{\left(2lm-2l\right)\sqrt{\left(5lm+2m-4)(5lm+2m-6\right)}}{2\sqrt{6}}\\ & + & \frac{\left(5l^{2}m^{2}+2lm^{2}-10lm-2m+5\right)}{5}\\ & + & \frac{\left(4lm-4m\right)\sqrt{\left(5lm+2m-5)(5lm+2m-6\right)}}{\sqrt{30}}. \end{array}$$

7. Let $BN_{\left(l,m\right)}$ be Borophene nanosheet. Then the reciprocal product temperature index is $\frac{\left(6m+12\right)}{\left(5lm+2m-3\right)}+\frac{2\left(4m-4\right)\sqrt{3}}{\sqrt{\left(5lm+2m-3)(5lm+2m-4\right)}}+\frac{\left(4l-4\right)\sqrt{15}}{\sqrt{\left(5lm+2m-3)(5lm+2m-5\right)}}+\frac{3\left(2l+4m\right)\sqrt{2}}{\sqrt{\left(5lm+2m-3)(5lm+2m-6\right)}}+\frac{4(lm-l)}{\left(5lm+2m-4\right)}+\frac{2\left(4lm-4l-4m+4\right)\sqrt{5}}{\sqrt{\left(5lm+2m-4)(5lm+2m-5\right)}}+\frac{2\left(2lm-2\right)\sqrt{6}}{\sqrt{\left(5lm+2m-4)(5lm+2m-6\right)}}+\frac{5(lm-m)}{\left(5lm+2m-5\right)}+\frac{\left(4lm-4m\right)\sqrt{30}}{\sqrt{\left(5lm+2m-5)(5lm+2m-6\right)}}.$

From Tables 3–5 along with Eq (6), we get

$$\begin{array}{ccc} RPT(BN_{\left(l,m\right)}) & = & \left(2m+4\right)\sqrt{\left[\frac{3}{(5lm+2m)-3}\times \frac{3}{(5lm+2m)-3}\right]}\\ & + & \left(4m-4\right)\sqrt{\left[\frac{3}{(5lm+2m)-3}\times \frac{4}{(5lm+2m)-4}\right]}\\ & + & \left(4l-4\right)\sqrt{\left[\frac{3}{(5lm+2m)-3}\times \frac{5}{(5lm+2m)-5}\right]}\\ & + & \left(2l+4m\right)\sqrt{\left[\frac{3}{(5lm+2m)-3}\times \frac{6}{(5lm+2m)-6}\right]}\\ & + & \left(lm-l\right)\sqrt{\left[\frac{4}{(5lm+2m)-4}\times \frac{4}{(5lm+2m)-4}\right]}\\ & + & \left(4lm-4l-4m+4\right)\sqrt{\left[\frac{4}{(5lm+2m)-4}\times \frac{5}{(5lm+2m)-5}\right]}\\ & + & \left(2lm-2\right)\sqrt{\left[\frac{4}{\left(5lm+2m-4\right)}\times \frac{6}{(5lm+2m)-6}\right]}\\ & + & \left(lm-m\right)\sqrt{\left[\frac{5}{(5lm+2m)-5}\times \frac{5}{(5lm+2m)-5}\right]}\\ & + & \left(4lm-4m\right)\sqrt{\left[\frac{5}{(5lm+2m)-5}\times \frac{6}{(5lm+2m)-6}\right]} \end{array}$$

After simplification, we get

$$\begin{array}{ccc} RPT(BN_{\left(l,m\right)}) & = & \frac{\left(6m+12\right)}{\left(5lm+2m-3\right)}\\ & + & \frac{2\left(4m-4\right)\sqrt{3}}{\sqrt{\left(5lm+2m-3)(5lm+2m-4\right)}}\\ & + & \frac{\left(4l-4\right)\sqrt{15}}{\sqrt{\left(5lm+2m-3)(5lm+2m-5\right)}}\\ & + & \frac{3\left(2l+4m\right)\sqrt{2}}{\sqrt{\left(5lm+2m-3)(5lm+2m-6\right)}}\\ & + & \frac{4(lm-l)}{\left(5lm+2m-4\right)}+\frac{2\left(4lm-4l-4m+4\right)\sqrt{5}}{\sqrt{\left(5lm+2m-4)(5lm+2m-5\right)}}\\ & + & \frac{2\left(2lm-2\right)\sqrt{6}}{\sqrt{\left(5lm+2m-4)(5lm+2m-6\right)}}+\frac{5(lm-m)}{\left(5lm+2m-5\right)}\\ & + & \frac{\left(4lm-4m\right)\sqrt{30}}{\sqrt{\left(5lm+2m-5)(5lm+2m-6\right)}} \end{array}$$

8. Let $BN_{\left(l,m\right)}$ be Borophene nanosheet. Then the F-temperature index is $\frac{18(2m+4)}{{\left(5lm+2m-3\right)}^{2}}+\frac{\left(4m-4\right)\left(625l^{2}m^{2}+500lm^{2}-840lm+100m^{2}-336m+288\right)}{{\left(5lm+2m-3\right)}^{2}{\left(5lm+2m-4\right)}^{2}}+\frac{2\left(4m-4\right)(425l^{2}m^{2}+340lm^{2}-600lm+68m^{2}-240m+225}{{\left(5lm+2m-3\right)}^{2}{\left(5lm+2m-5\right)}^{2}}+\frac{9\left(2l+4m\right)\left(125l^{2}m^{2}+100lm^{2}-180lm+20m^{2}-72m+72\right)}{{\left(5lm+2m-3\right)}^{2}{\left(5lm+2m-6\right)}^{2}}+\frac{32(lm-l)}{{\left(5lm+2m-4\right)}^{2}}+\frac{\left(4lm-4l-4m+4\right)\left(1025p^{2}m^{2}+820lm^{2}-1800lm+164m^{2}-720m+800\right)}{{\left(5lm+2m-4\right)}^{2}{\left(5lm+2m-5\right)}^{2}}+\frac{4\left(2lm-2l\right)\left(325l^{2}m^{2}+260lm^{2}-600lm+52m^{2}-240m+288\right)}{{\left(5lm+2m-4\right)}^{2}{\left(5lm+2m-6\right)}^{2}}+\frac{50(lm-m)}{{\left(5lm+2m-5\right)}^{2}}+\frac{\left(4lm-4m\right)\left(1525l^{2}m^{2}+1220lm^{2}-3300lm+244m^{2}-1320m+1800\right)}{{\left(5lm+2m-5\right)}^{2}{\left(5lm+2m-6\right)}^{2}}.$

From Tables 3–5 along with Eq (9), we have

$$\begin{array}{ccc} FT(BN_{\left(l,m\right)}) & = & \left(2m+4\right)[\{\frac{3}{(5lm+2m)-3}{\}}^{2}+\{\frac{3}{(5lm+2m)-3}{\}}^{2}]\\ & + & \left(4m-4\right)[\{\frac{3}{(5lm+2m)-3}{\}}^{2}+\{\frac{4}{(5lm+2m)-4}{\}}^{2}]\\ & + & \left(4l-4\right)[\{\frac{3}{(5lm+2m)-3}{\}}^{2}+\{\frac{5}{(5lm+2m)-5}{\}}^{2}]\\ & + & \left(2l+4m\right)[\{\frac{3}{(5lm+2m)-3}{\}}^{2}+\{\frac{6}{(5lm+2m)-6}{\}}^{2}]\\ & + & \left(lm-l\right)[\{\frac{4}{(5lm+2m)-4}{\}}^{2}+\{\frac{4}{(5lm+2m)-4}{\}}^{2}]\\ & + & \left(4lm-4l-4m-4\right)[\{\frac{4}{(5lm+2m)-4}{\}}^{2}+\{\frac{5}{(5lm+2m)-5}{\}}^{2}]\\ & + & \left(2lm-2l\right)[\{\frac{4}{(5lm+2m)-4}{\}}^{2}+\{\frac{6}{(5lm+2m)-6}{\}}^{2}]\\ & + & \left(lm-m\right)[\{\frac{5}{(5lm+2m)-5}{\}}^{2}+\{\frac{5}{(5lm+2m)-5}{\}}^{2}]\\ & + & \left(4lm-4m\right)[\{\frac{5}{(5lm+2m)-5}{\}}^{2}+\{\frac{5}{(5lm+2m)-6}{\}}^{2}] \end{array}$$

After simplification, we get

$$\begin{array}{ccc} FT(BN_{\left(l,m\right)}) & = & \frac{18(2m+4)}{{\left(5lm+2m-3\right)}^{2}}\\ & + & \frac{\left(4m-4\right)\left(625l^{2}m^{2}+500lm^{2}-840lm+100m^{2}-336m+288\right)}{{\left(5lm+2m-3\right)}^{2}{\left(5lm+2m-4\right)}^{2}}\\ & + & \frac{2\left(4m-4\right)(425l^{2}m^{2}+340lm^{2}-600lm+68m^{2}-240m+225}{{\left(5lm+2m-3\right)}^{2}{\left(5lm+2m-5\right)}^{2}}\\ & + & \frac{9\left(2l+4m\right)\left(125l^{2}m^{2}+100lm^{2}-180lm+20m^{2}-72m+72\right)}{{\left(5lm+2m-3\right)}^{2}{\left(5lm+2m-6\right)}^{2}}+\frac{32(lm-l)}{{\left(5lm+2m-4\right)}^{2}}\\ & + & \frac{\left(4lm-4l-4m+4\right)\left(1025p^{2}m^{2}+820lm^{2}-1800lm+164m^{2}-720m+800\right)}{{\left(5lm+2m-4\right)}^{2}{\left(5lm+2m-5\right)}^{2}}\\ & + & \frac{4\left(2lm-2l\right)\left(325l^{2}m^{2}+260lm^{2}-600lm+52m^{2}-240m+288\right)}{{\left(5lm+2m-4\right)}^{2}{\left(5lm+2m-6\right)}^{2}}+\frac{50(lm-m)}{{\left(5lm+2m-5\right)}^{2}}\\ & + & \frac{\left(4lm-4m\right)\left(1525l^{2}m^{2}+1220lm^{2}-3300lm+244m^{2}-1320m+1800\right)}{{\left(5lm+2m-5\right)}^{2}{\left(5lm+2m-6\right)}^{2}}. \end{array}$$

### 2.2 Comparative analysis of temperature indices for $BN_{\left(l,m\right)}$

**Fig 2:**
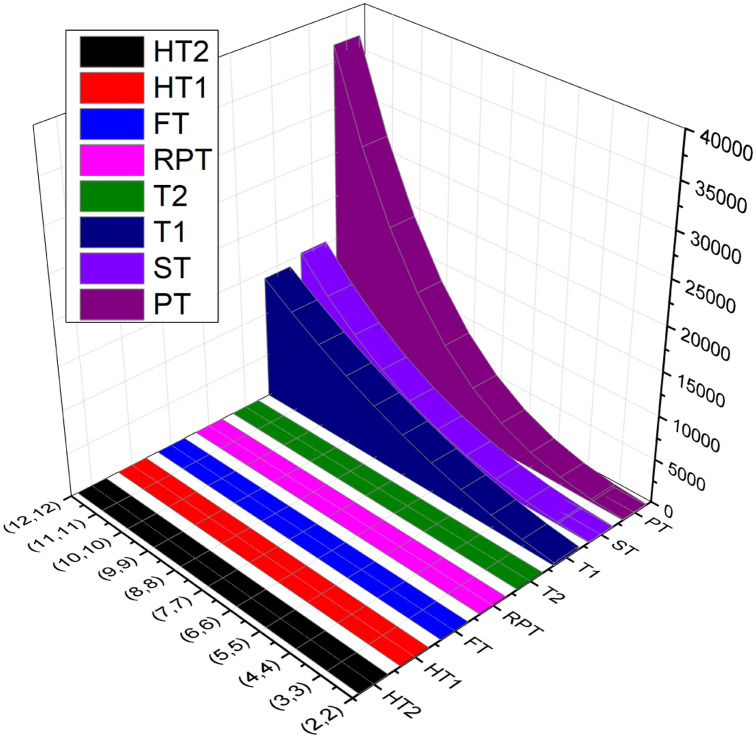
Graphical comparison of temperature indices of $BN_{\left(l,m\right)}$.

Numerical and graphical comparison of computed temperature indices of $BN_{\left(l,m\right)}$ for *l* = *m* is presented in this section. As the value of *l* or *m*, increases gradually, the value of temperatures indices *HT*_2_, *ST* and *PT* increases gradually while the numerical value of temperature indices *T*_1_, *T*_2_, *HT*_1_, *RPT* and *FT* decreases gradually. These changes are also represented in Table 6 and Fig 2.

**Table 6:** Numerical values of temperature indices of $BN_{\left(l,m\right)})$ for *l* = *m*.

(*l*, *m*)	*T*_1_	*T*_2_	*HT*_1_	*HT*_2_	*ST*	*PT*	*RPT*	*FT*
(2,2)	343.98	14.327	11.96	0.123	73.095	100.4	10.97	4.61
(3,3)	832.18	16.33	6.50	0.0105	247.34	361.34	10.80	2.09
(4,4)	1520.4	17.73	4.060	0.00194	582.06	904.18	10.86	1.2
(5,5)	2408.5	18.70	2.77	0.00053	1128.9	1865.6	10.95	0.80
(6,6)	3496.6	19.422	2.01	0.000185	1939.4	3406.1	11.03	0.56
(7,7)	4784.7	19.97	1.52	0.000075	3065.2	5710.6	11.099	0.42
(8,8)	6272.7	20.4	1.19	0.000034	4557.7	8987.6	11.15	0.33
(9,9)	7960.8	20.74	0.96	0.00017	6468.5	13470	11.20	0.26
(10,10)	9848.8	21.034	0.794	0.0000095	8849.0	19415	11.24	0.21
(11,11)	11937	21.27	0.665	0.0000054	11751	27100	11.28	0.18
(12,12)	14225	21.47	0.56	0.0000032	15226	36835	11.308	0.15

## 3 Borophene *β*_12_-sheets $BN_{\left(l,m\right)}$

**Fig 3:**
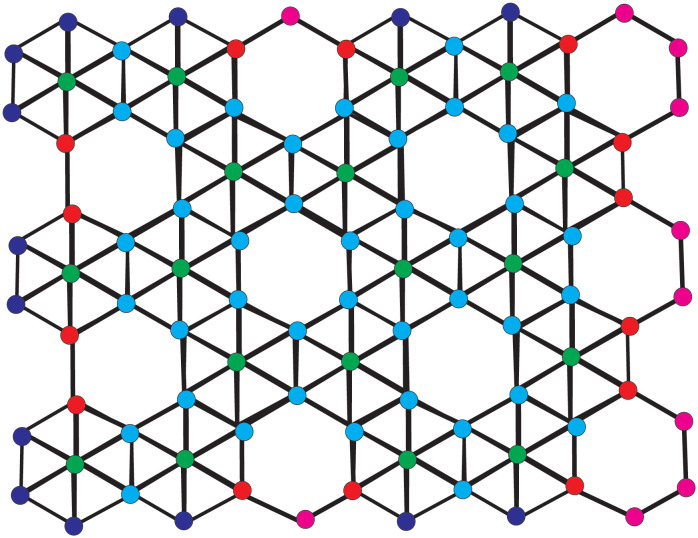
Borophene *β*_12_-sheets for *l* = 2 and *m* = 3.

Fig 3 is a graph borophene *β*_12_-sheet of for the particular values of *l* = 2 and *m* = 3. Here, in Borophene *β*_12_-sheet $BN_{\left(l,m\right)}$, there are eleven types of atom-bonds based on the valency of every atom of $BN_{\left(l,m\right)}$, which are (2 ∼ 2), (2 ∼ 4), (3 ∼ 3), (3 ∼ 4), (3 ∼ 5), (3 ∼ 6), (4 ∼ 4), (4 ∼ 5), (4 ∼ 6), (5 ∼ 5) and (5 ∼ 6) in $BN_{\left(l,m\right)}$. Based on valency, Tables 7 and 8 provide the partition of the atom-bonds of $BN_{\left(l,m\right)}$ shown as:

**Table 7:** Edge partition of Borophene *β*_12_-sheets $BN_{\left(l,m\right)}$.

Edge Partition	*E*_2∼2_	*E*_2∼4_	*E*_3∼3_	*E*_3∼4_	*E*_3∼5_	*E*_3∼6_
Cardinality	m+2	4l+2m-4	m+2	4l+2m-4	4l	4l+2m

**Table 8:** Edge partition of Borophene *β*_12_-sheets $BN_{\left(l,m\right)}$.

Edge Partition	*E*_4∼4_	*E*_4∼5_	*E*_4∼6_	*E*_5∼5_	*E*_5∼6_
Cardinality	m-1	4l+2m-4	4l+2m-4	18lm-7l-4m-5	24lm-32l-14m+24

The total number of atoms and atom-bonds $BN_{\left(l,m\right)}$:

$$\begin{array}{c} |V\left(BN_{\left(l,m\right)}\right)|=5lm+2m\;\;and\;\;|E\left(BN_{\left(l,m\right)}\right)|=42lm-15l-5m+6 \end{array}$$

By using Eq (1) and above partition of $BN_{\left(l,m\right)}$, as described in Tables 7 and 8.

### 3.1 Temperature indices for Borophene *β*_12_-sheet $BN_{\left(l,m\right)}$

9. Let $BN_{\left(l,m\right)}$ be a Borophene *β*_12_-sheet. Then the first temperature index of $BN_{\left(l,m\right)}$ is $\frac{4(m+2)}{5lm+2m-2}+\frac{\left(4l+2m-4)(30lm+12m-16\right)}{\left(5lm+2m-2)(5lm+2m-4\right)}+\frac{6(m+2)}{\left(5lm+2m-3\right)}+\frac{\left(4l+2m-4)(35lm+14m-24\right)}{\left(5lm+2m-3)(5lm+2m-4\right)}+\frac{4l(40lm+16m-30)}{\left(5lm+2m-3)(5lm+2m-5\right)}+\frac{\left(4l+2m)(45lm+18m-36\right)}{\left(5lm+2m-3)(5lm+2m-6\right)}+\frac{8(m-1)}{\left(5lm+2m-4\right)}+\frac{\left(4l+2m-4)(45lm+18m-40\right)}{\left(5lm+2m-4)(5lm+2m-5\right)}+\frac{\left(4l+2m-4)(50lm+20m-48\right)}{\left(5lm+2m-4)(5lm+2m-6\right)}+\frac{10(18lm-7l-4m-5)}{\left(5lm+2m-5\right)}+\frac{\left(24lm-32l+14m+24)(55lm+22m-60\right)}{\left(5lm+2m-5)(5lm+2m-6\right)}$.

Tables 9–12 along with Eq (2), gives

$$\begin{array}{ccc} T_{1}\left(BN_{\left(l,m\right)}\right) & = & \left(m+2\right)[\frac{2}{(5lm+2m)-2}+\frac{2}{(5lm+2m)-2}]\\ & + & \left(4l+2m-4\right)[\frac{2}{(5lm+2m)-2}+\frac{4}{(5lm+2m)-4}]\\ & + & \left(m+2\right)[\frac{3}{(5lm+2m)-3}+\frac{3}{(5lm+2m)-3}]\\ & + & \left(4l+2m-4\right)[\frac{3}{(5lm+2m)-3}+\frac{4}{(5lm+2m)-4}]\\ & + & \left(4l\right)[\frac{3}{(5lm+2m)-3}+\frac{5}{(5lm+2m)-5}]\\ & + & \left(4l+2m\right)[\frac{3}{(5lm+2m)-3}+\frac{6}{(5lm+2m)-6}]\\ & + & \left(m-1\right)[\frac{4}{(5lm+2m)-4}+\frac{4}{(5lm+2m)-4}]\\ & + & \left(4l+2m-4\right)[\frac{4}{(5lm+2m)-4}+\frac{5}{(5lm+2m)-5}]\\ & + & \left(4l+2m-4\right)[\frac{4}{(5lm+2m)-4}+\frac{6}{(5lm+2m)-6}]\\ & + & \left(18lm-7l-4m-5\right)[\frac{5}{(5lm+2m)-5}+\frac{5}{(5lm+2m)-5}]\\ & + & \left(24lm-32l-14m+24\right)[\frac{5}{(5lm+2m)-5}+\frac{6}{(5lm+2m)-6}] \end{array}$$

**Table 9:** Atomic-bond partition of $BN_{\left(l,m\right)}$.

(*T*_*u*_, *T*_*v*_)	$\left(\frac{2}{\left(5lm+2m-2\right)},\frac{2}{\left(5lm+2m-2\right)}\right)$	$\left(\frac{2}{\left(5lm+2m-2\right)},\frac{4}{\left(5lm+2m-4\right)}\right)$	$\left(\frac{3}{\left(5lm+2m-3\right)},\frac{3}{\left(5lm+2m-3\right)}\right)$
Frequency	*m* + 2	4*l* + 2*m* − 4	*m* + 2

**Table 10:** Atomic-bond partition of $BN_{\left(l,m\right)}$.

(*T*_*u*_, *T*_*v*_)	$\left(\frac{3}{\left(5lm+2m-3\right)},\frac{4}{\left(5lm+2m-4\right)}\right)$	$\left(\frac{3}{\left(5lm+2m-3\right)},\frac{5}{\left(5lm+2m-5\right)}\right)$	$\left(\frac{3}{\left(5lm+2m-3\right)},\frac{6}{\left(5lm+2m-6\right)}\right)$
Frequency	4*l* + 2*m* − 4	4*l*	4*l* + 2*m*

**Table 11:** Atomic-bond partition of $BN_{\left(l,m\right)}$.

(*T*_*u*_, *T*_*v*_)	$\left(\frac{4}{\left(5lm+2m-4\right)},\frac{4}{\left(5lm+2m-4\right)}\right)$	$\left(\frac{4}{\left(5lm+2m-4\right)},\frac{5}{\left(5lm+2m-5\right)}\right)$	$\left(\frac{4}{\left(5lm+2m-4\right)},\frac{6}{\left(5lm+2m-6\right)}\right)$
Frequency	*m* − 1	4*l* + 2*m* − 4	4*l* + 2*m* − 4

**Table 12:** Atomic-bond partition of $BN_{\left(l,m\right)}$.

(*T*_*u*_, *T*_*v*_)	$\left(\frac{5}{\left(5lm+2m-5\right)},\frac{5}{\left(5lm+2m-5\right)}\right)$	$\left(\frac{6}{\left(5lm+2m-6\right)},\frac{5}{\left(5lm+2m-6\right)}\right)$
Frequency	18*lm* − 7*l* − 4*m* − 5	24*lm* − 32*l* − 14*m* + 24

After simplification, we get

$$\begin{array}{ccc} T_{1}\left(BN_{\left(l,m\right)}\right) & = & \frac{4(m+2)}{5lm+2m-2}+\frac{\left(4l+2m-4)(30lm+12m-16\right)}{\left(5lm+2m-2)(5lm+2m-4\right)}\\ & + & \frac{6(m+2)}{\left(5lm+2m-3\right)}+\frac{\left(4l+2m-4)(35lm+14m-24\right)}{\left(5lm+2m-3)(5lm+2m-4\right)}\\ & + & \frac{4l(40lm+16m-30)}{\left(5lm+2m-3)(5lm+2m-5\right)}+\frac{\left(4l+2m)(45lm+18m-36\right)}{\left(5lm+2m-3)(5lm+2m-6\right)}\\ & + & \frac{8(m-1)}{\left(5lm+2m-4\right)}+\frac{\left(4l+2m-4)(45lm+18m-40\right)}{\left(5lm+2m-4)(5lm+2m-5\right)}\\ & + & \frac{\left(4l+2m-4)(50lm+20m-48\right)}{\left(5lm+2m-4)(5lm+2m-6\right)}+\frac{10(18lm-7l-4m-5)}{\left(5lm+2m-5\right)}\\ & + & \frac{\left(24lm-32l+14m+24)(55lm+22m-60\right)}{\left(5lm+2m-5)(5lm+2m-6\right)}. \end{array}$$

10. Let $BN_{\left(l,m\right)}$ be a Borophene *β*_12_-sheet. Then the second temperature index is $\frac{4(m+2)}{{\left(5lm+2m-2\right)}^{2}}+\frac{8(4l+2m-4)}{\left(5lm+2m-2)(5lm+2m-4\right)}+\frac{9(m+2)}{{\left(5lm+2m-3\right)}^{2}}+\frac{12(4l+2m-4)}{\left(5lm+2m-3)(5lm+2m-4\right)}+\frac{15(4l)}{\left(5lm+2m-3)(5lm+2m-5\right)}+\frac{18(4l+2m)}{\left(5lm+2m-3)(5lm+2m-6\right)}+\frac{16(m-1)}{{\left(5lm+2m-4\right)}^{2}}+\frac{20(4l+2m-4)}{\left(5lm+2m-4)(5lm+2m-5\right)}+\frac{24(4l+2m-4)}{\left(5lm+2m-4)(5lm+2m-6\right)}+\frac{25(18lm-7l-4m-5)}{{\left(5lm+2m-5\right)}^{2}}+\frac{30(24lm-32l-14m+24)}{\left(5lm+2m-5)(5lm+2m-6\right)}$.

Tables 9–12 along with Eq (3), implies

$$\begin{array}{ccc} T_{2}\left(BN_{\left(l,m\right)}\right) & = & \left(m+2\right)[\frac{2}{(5lm+2m)-2}\times \frac{2}{(5lm+2m)-2}]\\ & + & \left(4l+2m-4\right)[\frac{2}{(5lm+2m)-2}\times \frac{4}{(5lm+2m)-4}]\\ & + & \left(m+2\right)[\frac{3}{(5lm+2m)-3}\times \frac{3}{(5lm+2m)-3}]\\ & + & \left(4l+2m-4\right)[\frac{3}{(5lm+2m)-3}\times \frac{4}{(5lm+2m)-4}]\\ & + & \left(4l\right)[\frac{3}{(5lm+2m)-3}\times \frac{5}{(5lm+2m)-5}]\\ & + & \left(4l+2m\right)[\frac{3}{(5lm+2m)-3}\times \frac{6}{(5lm+2m)-6}]\\ & + & \left(m-1\right)[\frac{4}{(5lm+2m)-4}\times \frac{4}{(5lm+2m)-4}]\\ & + & \left(4l+2m-4\right)[\frac{4}{(5lm+2m)-4}\times \frac{5}{(5lm+2m)-5}]\\ & + & \left(4l+2m-4\right)[\frac{4}{(5lm+2m)-4}\times \frac{6}{(5lm+2m)-6}]\\ & + & \left(18lm-7l-4m-5\right)[\frac{5}{(5lm+2m)-5}\times \frac{5}{(5lm+2m)-5}]\\ & + & \left(24lm-32l-14m+24\right)[\frac{5}{(5lm+2m)-5}\times \frac{6}{(5lm+2m)-6}] \end{array}$$

After simplification, we get

$$\begin{array}{ccc} T_{2}\left(BN_{\left(l,m\right)}\right) & = & \frac{4(m+2)}{{\left(5lm+2m-2\right)}^{2}}+\frac{8(4l+2m-4)}{\left(5lm+2m-2)(5lm+2m-4\right)}\\ & + & \frac{9(m+2)}{{\left(5lm+2m-3\right)}^{2}}+\frac{12(4l+2m-4)}{\left(5lm+2m-3)(5lm+2m-4\right)}\\ & + & \frac{15(4l)}{\left(5lm+2m-3)(5lm+2m-5\right)}\\ & + & \frac{18(4l+2m)}{\left(5lm+2m-3)(5lm+2m-6\right)}+\frac{16(m-1)}{{\left(5lm+2m-4\right)}^{2}}\\ & + & \frac{20(4l+2m-4)}{\left(5lm+2m-4)(5lm+2m-5\right)}+\frac{24(4l+2m-4)}{\left(5lm+2m-4)(5lm+2m-6\right)}\\ & + & \frac{25(18lm-7l-4m-5)}{{\left(5lm+2m-5\right)}^{2}}+\frac{30(24lm-32l-14m+24)}{\left(5lm+2m-5)(5lm+2m-6\right)}. \end{array}$$

11. Let $BN_{\left(l,m\right)}$ be Borophene *β*_12_-sheet. Then the first hyper temperature index is $\frac{16(m+2)}{{\left(5lm+2m-2\right)}^{2}}+\frac{4\left(4l+2m-4\right){\left(15lm+6m-8\right)}^{2})}{{\left(5lm+2m-2\right)}^{2}{\left(5lm+2m-4\right)}^{2}}+\frac{36(m+2)}{{\left(5lm+2m-3\right)}^{2}}+\frac{\left(4l+2m-4\right){\left(35lm+14m-24\right)}^{2}}{{\left(5lm+2m-3\right)}^{2}{\left(5lm+2m-4\right)}^{2}}+\frac{4\left(4l\right){\left(20lm+8m-15\right)}^{2}}{{\left(5lm+2m-3\right)}^{2}{\left(5lm+2m-5\right)}^{2}}+\frac{81\left(4l+2m\right){\left(5lm+2m-4\right)}^{2}}{{\left(5lm+2m-3\right)}^{2}{\left(5lm+2m-6\right)}^{2}}+\frac{64(m-1)}{{\left(5lm+2m-4\right)}^{2}}+\frac{\left(4l+2m-4\right){\left(45lm+18m-40\right)}^{2}}{{\left(5lm+2m-4\right)}^{2}{\left(5lm+2m-5\right)}^{2}}+\frac{4\left(4l+2m-4\right){\left(25lm+10m-24\right)}^{2}}{{\left(5lm+2m-4\right)}^{2}{\left(5lm+2m-6\right)}^{2}}+\frac{100{\left(18lm-7l-4m-5\right)}^{2}}{\left(5lm+2m-5\right)}+\frac{\left(24lm-32l-14m+24\right){\left(55lm+22m-60\right)}^{2}}{{\left(5lm+2m-5\right)}^{2}{\left(5lm+2m-6\right)}^{2}}$.

From Tables 9–12 along with Eq (4), we obtain

$$\begin{array}{ccc} HT_{1}\left(BN_{\left(l,m\right)}\right) & = & \left(m+2\right){\left[\frac{2}{(5lm+2m)-2}+\frac{2}{(5lm+2m)-2}\right]}^{2}\\ & + & \left(4l+2m-4\right){\left[\frac{2}{(5lm+2m)-2}+\frac{4}{(5lm+2m)-4}\right]}^{2}\\ & + & \left(m+2\right){\left[\frac{3}{(5lm+2m)-3}+\frac{3}{(5lm+2m)-3}\right]}^{2}\\ & + & \left(4l+2m-4\right){\left[\frac{3}{(5lm+2m)-3}+\frac{4}{(5lm+2m)-4}\right]}^{2}\\ & + & \left(4l\right){\left[\frac{3}{(5lm+2m)-3}+\frac{5}{(5lm+2m)-5}\right]}^{2}\\ & + & \left(4l+2m\right){\left[\frac{3}{(5lm+2m)-3}+\frac{6}{(5lm+2m)-6}\right]}^{2}\\ & + & \left(m-1\right){\left[\frac{4}{(5lm+2m)-4}+\frac{4}{(5lm+2m)-4}\right]}^{2}\\ & + & \left(4l+2m-4\right){\left[\frac{4}{(5lm+2m)-4}+\frac{5}{(5lm+2m)-5}\right]}^{2}\\ & + & \left(4l+2m-4\right){\left[\frac{4}{(5lm+2m)-4}+\frac{6}{(5lm+2m)-6}\right]}^{2}\\ & + & \left(18lm-7l-4m-5\right){\left[\frac{5}{(5lm+2m)-5}+\frac{5}{(5lm+2m)-5}\right]}^{2}\\ & + & \left(24lm-32l-14m+24\right){\left[\frac{5}{(5lm+2m)-5}+\frac{6}{(5lm+2m)-6}\right]}^{2} \end{array}$$

After simplification, we get

$$\begin{array}{ccc} HT_{1}\left(BN_{\left(l,m\right)}\right) & = & \frac{16(m+2)}{{\left(5lm+2m-2\right)}^{2}}+\frac{4\left(4l+2m-4\right){\left(15lm+6m-8\right)}^{2}}{{\left(5lm+2m-2\right)}^{2}{\left(5lm+2m-4\right)}^{2}}\\ & + & \frac{36(m+2)}{{\left(5lm+2m-3\right)}^{2}}+\frac{\left(4l+2m-4\right){\left(35lm+14m-24\right)}^{2}}{{\left(5lm+2m-3\right)}^{2}{\left(5lm+2m-4\right)}^{2}}\\ & + & \frac{4\left(4l\right){\left(20lm+8m-15\right)}^{2}}{{\left(5lm+2m-3\right)}^{2}{\left(5lm+2m-5\right)}^{2}}\\ & + & \frac{81\left(4l+2m\right){\left(5lm+2m-4\right)}^{2}}{{\left(5lm+2m-3\right)}^{2}{\left(5lm+2m-6\right)}^{2}}+\frac{64(m-1)}{{\left(5lm+2m-4\right)}^{2}}\\ & + & \frac{\left(4l+2m-4\right){\left(45lm+18m-40\right)}^{2}}{{\left(5lm+2m-4\right)}^{2}{\left(5lm+2m-5\right)}^{2}}\\ & + & \frac{4\left(4l+2m-4\right){\left(25lm+10m-24\right)}^{2}}{{\left(5lm+2m-4\right)}^{2}{\left(5lm+2m-6\right)}^{2}}+\frac{100{\left(18lm-7l-4m-5\right)}^{2}}{{\left(5lm+2m-5\right)}^{2}}\\ & + & \frac{\left(24lm-32l-14m+24\right){\left(55lm+22m-60\right)}^{2}}{{\left(5lm+2m-5\right)}^{2}{\left(5lm+2m-6\right)}^{2}}. \end{array}$$

12. Let $BN_{\left(l,m\right)}$ be Borophene *β*_12_-sheet. Then the second hyper temperature index is $\frac{16(m+2)}{{\left(5lm+2m-2\right)}^{4}}+\frac{64(4l+2m-4)}{{\left(5lm+2m-2\right)}^{2}{\left(5lm+2m-4\right)}^{2}}+\frac{81(m+2)}{{\left(5lm+2m-3\right)}^{4}}+\frac{144(4l+2m-4)}{{\left(5lm+2m-3\right)}^{2}{\left(5lm+2m-4\right)}^{2}}+\frac{225(4l)}{{\left(5lm+2m-3\right)}^{2}{\left(5lm+2m-5\right)}^{2}}+\frac{324(4l+2m)}{{\left(5lm+2m-3\right)}^{2}{\left(5lm+2m-6\right)}^{2}}+\frac{256(m-1)}{{\left(5lm+2m-4\right)}^{4}}+\frac{400(4l+2m-4)}{{\left(5lm+2m-4\right)}^{2}{\left(5lm+2m-5\right)}^{2}}+\frac{576(4l+2m-4)}{{\left(5lm+2m-4\right)}^{2}{\left(5lm+2m-6\right)}^{2}}+\frac{625(18lm-7l-4m-5)}{{\left(5lm+2m-5\right)}^{4}}+\frac{900(24lm-32l-14m+24)}{{\left(5lm+2m-5\right)}^{2}{\left(5lm+2m-6\right)}^{2}}.$

From Tables 9–12 along with Eq (5), we get

$$\begin{array}{ccc} HT_{2}\left(BN_{\left(l,m\right)}\right) & = & \left(m+2\right){\left[\frac{2}{(5lm+2m)-2}\times \frac{2}{(5lm+2m)-2}\right]}^{2}\\ & + & \left(4l+2m-4\right){\left[\frac{2}{(5lm+2m)-2}\times \frac{4}{(5lm+2m)-4}\right]}^{2}\\ & + & \left(m+2\right){\left[\frac{3}{(5lm+2m)-3}\times \frac{3}{(5lm+2m)-3}\right]}^{2}\\ & + & \left(4l+2m-4\right){\left[\frac{3}{(5lm+2m)-3}\times \frac{4}{(5lm+2m)-4}\right]}^{2}\\ & + & \left(4l\right){\left[\frac{3}{(5lm+2m)-3}\times \frac{5}{(5lm+2m)-5}\right]}^{2}\\ & + & \left(4l+2m\right){\left[\frac{3}{(5lm+2m)-3}\times \frac{6}{(2lm+2m)-6}\right]}^{2}\\ & + & \left(m-1\right){\left[\frac{4}{(5lm+2m)-4}\times \frac{4}{(5lm+2m)-4}\right]}^{2}\\ & + & \left(4l+2m-4\right){\left[\frac{4}{(5lm+2m)-4}\times \frac{5}{(5lm+2m)-5}\right]}^{2}\\ & + & \left(4l+2m-4\right){\left[\frac{4}{(5lm+2m)-4}\times \frac{6}{(2lm+2m)-6}\right]}^{2}\\ & + & \left(18lm-7l-4m-5\right){\left[\frac{5}{(5lm+2m)-5}\times \frac{5}{(2lm+2m)-5}\right]}^{2}\\ & + & \left(24lm-32l-14m+24\right){\left[\frac{5}{(5lm+2m)-5}\times \frac{6}{(2lm+2m)-6}\right]}^{2} \end{array}$$

After simplification, we get

$$\begin{array}{ccc} HT_{2}\left(BN_{\left(l,m\right)}\right) & = & \frac{16(m+2)}{{\left(5lm+2m-2\right)}^{4}}+\frac{64(4l+2m-4)}{{\left(5lm+2m-2\right)}^{2}{\left(5lm+2m-4\right)}^{2}}\\ & + & \frac{81(m+2)}{{\left(5lm+2m-3\right)}^{4}}+\frac{144(4l+2m-4)}{{\left(5lm+2m-3\right)}^{2}{\left(5lm+2m-4\right)}^{2}}\\ & + & \frac{225(4l)}{{\left(5lm+2m-3\right)}^{2}{\left(5lm+2m-5\right)}^{2}}\\ & + & \frac{324(4l+2m)}{{\left(5lm+2m-3\right)}^{2}{\left(5lm+2m-6\right)}^{2}}+\frac{256(m-1)}{{\left(5lm+2m-4\right)}^{4}}\\ & + & \frac{400(4l+2m-4)}{{\left(5lm+2m-4\right)}^{2}{\left(5lm+2m-5\right)}^{2}}\\ & + & \frac{576(4l+2m-4)}{{\left(5lm+2m-4\right)}^{2}{\left(5lm+2m-6\right)}^{2}}\\ & + & \frac{625(18lm-7l-4m-5)}{{\left(5lm+2m-5\right)}^{4}}+\frac{900(24lm-32l-14m+24)}{{\left(5lm+2m-5\right)}^{2}{\left(5lm+2m-6\right)}^{2}}. \end{array}$$

13. Let $BN_{\left(l,m\right)}$ be Borophene *β*_12_-sheet. Then the sum connectivity temperature index is $\frac{\left(m+2\right)\sqrt{\left(5lm+2m-2\right)}}{2}+\frac{\left(4l+2m-4\right)\sqrt{\left(5lm+2m-2)(5lm+2m-4\right)}}{\sqrt{30lm+12m-16}}+\frac{\left(m+2\right)\sqrt{\left(5lm+2m-3\right)}}{\sqrt{6}}+\frac{\sqrt{\left(5lm+2m-3)(5lm+2m-4\right)}}{\sqrt{35lm+14m-24)}}+\frac{4l\sqrt{\left(5lm+2m-3)(5lm+2m-5\right)}}{\sqrt{40lm+16m-30}}+\frac{\left(4l+2m\right)\sqrt{\left(5lm+2m-3)(5lm+2m-6\right)}}{3\sqrt{5lm+2m-4}}+\frac{\left(m-1\right)\sqrt{\left(5lm+2m-4\right)}}{2\sqrt{2}}+\frac{\left(4l+2m-4\right)\sqrt{\left(5lm+2m-4)(5lm+2m-5\right)}}{\sqrt{45lm+18m-40}}+\frac{\left(4l+2m-4\right)\sqrt{\left(5lm+2m-4)(5lm+2m-6\right)}}{\sqrt{50lm+20m-48}}+\frac{\left(18lm-7l-4m-5\right)\sqrt{\left(5lm+2m-5\right)}}{\sqrt{10}}+\frac{\left(24lm-32l-14m+24\right)\sqrt{(5lm+2m-5)(5lm+2m-6}}{\sqrt{55lm-22m-60}}$.

From Tables 9–12 along with Eq (8), we have

$$\begin{array}{ccc} ST(BN_{\left(l,m\right)}) & = & \left(m+2\right)\frac{1}{\sqrt{\left[\frac{2}{(5lm+2m)-2}+\frac{2}{(5lm+2m)-2}\right]}}\\ & + & \left(4l+2m-4\right)\frac{1}{\sqrt{\left[\frac{2}{(5lm+2m)-2}+\frac{4}{(5lm+2m)-4}\right]}}\\ & + & \left(m+2\right)\frac{1}{\sqrt{\left[\frac{3}{(5lm+2m)-3}+\frac{3}{(5lm+2m)-3}\right]}}\\ & + & \left(4l+2m-4\right)\frac{1}{\sqrt{\left[\frac{3}{(5lm+2m)-3}+\frac{4}{(5lm+2m)-4}\right]}}\\ & + & \left(4l\right)\frac{1}{\sqrt{\left[\frac{3}{(5lm+2m)-3}+\frac{5}{(5lm+2m)-5}\right]}}\\ & + & \left(4l+2m\right)\frac{1}{\sqrt{\left[\frac{3}{(5lm+2m)-3}+\frac{6}{(5lm+2m)-6}\right]}}\\ & + & \left(m-1\right)\frac{1}{\sqrt{\left[\frac{4}{(5lm+2m)-4}+\frac{4}{(5lm+2m)-4}\right]}}\\ & + & \left(4l+2m-4\right)\frac{1}{\sqrt{\left[\frac{4}{(5lm+2m)-4}+\frac{5}{(5lm+2m)-5}\right]}}\\ & + & \left(4l+2m-4\right)\frac{1}{\sqrt{\left[\frac{4}{(5lm+2m)-4}+\frac{6}{(5lm+2m)-6}\right]}}\\ & + & \left(18lm-7l-4m-5\right)\frac{1}{\sqrt{\left[\frac{5}{(5lm+2m)-5}+\frac{5}{(5lm+2m)-5}\right]}}\\ & + & \left(24lm-32l-14m+24\right)\frac{1}{\sqrt{\left[\frac{5}{(5lm+2m)-5}+\frac{6}{(5lm+2m)-6}\right]}} \end{array}$$

After simplification, we get

$$\begin{array}{ccc} ST(BN_{\left(l,m\right)}) & = & \frac{\left(m+2\right)\sqrt{\left(5lm+2m-2\right)}}{2}\\ & + & \frac{\left(4l+2m-4\right)\sqrt{\left(5lm+2m-2)(5lm+2m-4\right)}}{\sqrt{30lm+12m-16}}\\ & + & \frac{\left(m+2\right)\sqrt{\left(5lm+2m-3\right)}}{\sqrt{6}}\\ & + & \frac{\sqrt{(5lm+2m-3)(5lm+2m-4}}{\sqrt{35lm+14m-24}}\\ & + & \frac{4l\sqrt{\left(5lm+2m-3)(5lm+2m-5\right)}}{\sqrt{40lm+16m-30}}\\ & + & \frac{\left(4l+2m\right)\sqrt{\left(5lm+2m-3)(5lm+2m-6\right)}}{3\sqrt{5lm+2m-4}}\\ & + & \frac{\left(m-1\right)\sqrt{\left(5lm+2m-4\right)}}{2\sqrt{2}}\\ & + & \frac{\left(4l+2m-4\right)\sqrt{\left(5lm+2m-4)(5lm+2m-5\right)}}{\sqrt{45lm+18m-40}}\\ & + & \frac{\left(4l+2m-4\right)\sqrt{\left(5lm+2m-4)(5lm+2m-6\right)}}{\sqrt{50lm+20m-48}}\\ & + & \frac{\left(18lm-7l-4m-5\right)\sqrt{\left(5lm+2m-5\right)}}{\sqrt{10}}\\ & + & \frac{\left(24lm-32l-14m+24\right)\sqrt{(5lm+2m-5)(5lm+2m-6}}{\sqrt{55lm-22m-60}}. \end{array}$$

14. Let $BN_{\left(l,m\right)}$ be Borophene *β*_12_-sheet. Then the product connectivity temperature index is $\frac{\left(m+2)(5lm+2m-2\right)}{2}+\frac{\left(4l+2m-4\right)\sqrt{\left(5lm+2m-2)(5lm+2m-4\right)}}{2\sqrt{2}}+\frac{(m+2)\left(5lm+2m-3\right)}{3}+\frac{\left(4l+2m-4\right)\sqrt{\left(5lm+2m-3)(5lm+2m-4\right)}}{2\sqrt{3}}+\frac{\left(4l\right)\sqrt{\left(5lm+2m-3)(5lm+2m-5\right)}}{\sqrt{15}}+\frac{\left(4l+2m\right)\sqrt{\left(5lm+2m-3)(5lm+2m-6\right)}}{3\sqrt{2}}+\frac{(m-1)\left(5lm+2m-4\right)}{4}+\frac{\left(4l+2m-4\right)\sqrt{\left(5lm+2m-4)(5lm+2m-5\right)}}{2\sqrt{\left(5\right)}}+\frac{\left(4l+2m-4\right)\sqrt{\left(5lm+2m-4)(5lm+2m-6\right)}}{2\sqrt{6}}+\frac{(18lm-7l-4m-5)\left((5lm+2m-5\right)}{\left(5\right)}+\frac{\left(24lm-32l-14m+24\right)\sqrt{\left(5lm+2m-5)(5lm+2m-6\right)}}{\sqrt{30}}.$

From Tables 9–12 along with Eq (7), we obtain

$$\begin{array}{ccc} PT(BN_{\left(l,m\right)}) & = & \left(m+2\right)\frac{1}{\sqrt{\left[\frac{2}{(5lm+2m)-2}\times \frac{2}{(5lm+2m)-2}\right]}}\\ & + & \left(4l+2m-4\right)\frac{1}{\sqrt{\left[\frac{2}{(5lm+2m)-2}\times \frac{4}{(5lm+2m)-4}\right]}}\\ & + & \left(m+2\right)\frac{1}{\sqrt{\left[\frac{3}{(5lm+2m-3}\times \frac{3}{\left(5lm+2m-3\right)}\right]}}\\ & + & \left(4l+2m-4\right)\frac{1}{\sqrt{\left[\frac{3}{(5lm+2m)-3}\times \frac{4}{(5lm+2m)-4}\right]}}\\ & + & \left(4l\right)\frac{1}{\sqrt{\left[\frac{3}{(5lm+2m)-3}\times \frac{5}{(5lm+2m)-5}\right]}}\\ & + & \left(4l+2m\right)\frac{1}{\sqrt{\left[\frac{3}{(5lm+2m)-3}\times \frac{6}{(5lm+2m)-6}\right]}}\\ & + & \left(m-1\right)\frac{1}{\sqrt{\left[\frac{4}{(5lm+2m)-4}\times \frac{4}{(5lm+2m)-4}\right]}}\\ & + & \left(4l+2m-4\right)\frac{1}{\sqrt{\left[\frac{4}{(5lm+2m)-4}\times \frac{5}{(5lm+2m)-5}\right]}}\\ & + & \left(4l+2m-4\right)\frac{1}{\sqrt{\left[\frac{4}{(5lm+2m)-4}\times \frac{6}{(5lm+2m)-6}\right]}}\\ & + & \left(18lm-7l-4m-5\right)\frac{1}{\sqrt{\left[\frac{5}{(5lm+2m)-5}\times \frac{5}{(5lm+2m)-5}\right]}}\\ & + & \left(24lm-32l-14m+24\right)\frac{1}{\sqrt{\left[\frac{5}{(5lm+2m)-5}\times \frac{6}{(5lm+2m)-6}\right]}} \end{array}$$

After simplification, we get

$$\begin{array}{ccc} PT(BN_{\left(l,m\right)}) & = & \frac{\left(m+2)(5lm+2m-2\right)}{2}\\ & + & \frac{\left(4l+2m-4\right)\sqrt{\left(5lm+2m-2)(5lm+2m-4\right)}}{2\sqrt{2}}\\ & + & \frac{(m+2)\left(5lm+2m-3\right)}{3}\\ & + & \frac{\left(4l+2m-4\right)\sqrt{\left(5lm+2m-3)(5lm+2m-4\right)}}{2\sqrt{3}}\\ & + & \frac{\left(4l\right)\sqrt{\left(5lm+2m-3)(5lm+2m-5\right)}}{\sqrt{15}}\\ & + & \frac{\left(4l+2m\right)\sqrt{\left(5lm+2m-3)(5lm+2m-6\right)}}{3\sqrt{2}}\\ & + & \frac{(m-1)\left(5lm+2m-4\right)}{4}\\ & + & \frac{\left(4l+2m-4\right)\sqrt{\left(5lm+2m-4)(5lm+2m-5\right)}}{2\sqrt{\left(5\right)}}\\ & + & \frac{\left(4l+2m-4\right)\sqrt{\left(5lm+2m-4)(5lm+2m-6\right)}}{2\sqrt{6}}\\ & + & \frac{(18lm-7l-4m-5)\left((5lm+2m-5\right)}{\left(5\right)}\\ & + & \frac{\left(24lm-32l-14m+24\right)\sqrt{\left(5lm+2m-5)(5lm+2m-6\right)}}{\sqrt{30}}. \end{array}$$

15. Let $BN_{\left(l,m\right)}$ be Borophene *β*_12_-sheet. Then the reciprocal product temperature index is
  $$\begin{array}{ccc} RPT(BN_{\left(l,m\right)}) & = & \frac{2(m+2)}{\left(5lm+2m-2\right)}\\ & + & \frac{2\left(4l+2m-4\right)\sqrt{2}}{\sqrt{\left(5lm+2m-2)(5lm+2m-4\right)}}\\ & + & \frac{9(4l+2m)}{\left(5lm+2m-3\right)}+\frac{2\left(4l+2m-4\right)\sqrt{3}}{\sqrt{\left(5lm+2m-3)(5lm+2m-4\right)}}\\ & + & \frac{4l\sqrt{15}}{\sqrt{(}5lm+2m-3)\left(5lm+2m-5\right)}\\ & + & \frac{3\left(4l+2m\right)\sqrt{2}}{\sqrt{\left(5lm+2m-3)(5lm+2m-6\right)}}\\ & + & \frac{4(m-1)}{\left(5lm+2m-4\right)}\\ & + & \frac{2\left(4l+2m-4\right)\sqrt{5}}{\sqrt{(}5lm+2m-4)\left(5lm+2m-5\right)}\\ & + & \frac{2\left(4l+2m-4\right)\sqrt{6}}{\sqrt{\left(5lm+2m-4)(5lm+2m-6\right)}}\\ & + & \frac{5(18lm-7l-4m-5)}{\left(5lm+2m-5\right)}+\frac{\left(24lm-32l-14m+24\right)\sqrt{30}}{\sqrt{\left(5lm+2m-5)(5lm+2m-6\right)}}. \end{array}$$

From Tables 9–12 along with Eq (6), we attain

$$\begin{array}{ccc} RPT(BN_{\left(l,m\right)}) & = & \left(m+2\right)\sqrt{\left[\frac{2}{(5lm+2m)-2}\times \frac{2}{(5lm+2m)-2}\right]}\\ & + & \left(4l+2m-4\right)\sqrt{\left[\frac{2}{(5lm+2m)-2}\times \frac{4}{(5lm+2m)-4}\right]}\\ & + & \left(4l+2m\right)\sqrt{\left[\frac{3}{(5lm+2m-3)-3}\times \frac{3}{(5lm+2m)-3}\right]}\\ & + & \left(4l+2m-4\right)\sqrt{\left[\frac{3}{(5lm+2m-3)-3}\times \frac{4}{(5lm+2m)-4}\right]}\\ & + & \left(4l\right)\sqrt{\left[\frac{3}{(5lm+2m)-3}\times \frac{5}{(5lm+2m)-5}\right]}\\ & + & \left(4l+2m\right)\sqrt{\left[\frac{3}{(5lm+2m)-3}\times \frac{6}{(5lm+2m)-6}\right]}\\ & + & \left(m-1\right)\sqrt{\left[\frac{4}{\left(5lm+2m-4\right)}\times \frac{4}{(5lm+2m)-4}\right]}\\ & + & \left(4l+2m-4\right)\sqrt{\left[\frac{4}{(5lm+2m)-4}\times \frac{5}{(5lm+2m)-5}\right]}\\ & + & \left(4l+2m-4\right)\sqrt{\left[\frac{4}{(5lm+2m)-4}\times \frac{6}{(5lm+2m)-6}\right]}\\ & + & \left(18lm-7l-4m-5\right)\sqrt{\left[\frac{5}{(5lm+2m)-5}\times \frac{5}{(5lm+2m)-5}\right]}\\ & + & \left(24lm-32l-14m+24\right)\sqrt{\left[\frac{5}{(5lm+2m)-5}\times \frac{6}{(5lm+2m)-6}\right]} \end{array}$$

After simplification, we get

$$\begin{array}{ccc} RPT(BN_{\left(l,m\right)}) & = & \frac{2(m+2)}{\left(5lm+2m-2\right)}\\ & + & \frac{2\left(4l+2m-4\right)\sqrt{2}}{\sqrt{\left(5lm+2m-2)(5lm+2m-4\right)}}+\frac{9(4l+2m)}{\left(5lm+2m-3\right)}\\ & + & \frac{2\left(4l+2m-4\right)\sqrt{3}}{\sqrt{\left(5lm+2m-3)(5lm+2m-4\right)}}\\ & + & \frac{4l\sqrt{15}}{\sqrt{(}5lm+2m-3)\left(5lm+2m-5\right)}\\ & + & \frac{3\left(4l+2m\right)\sqrt{2}}{\sqrt{\left(5lm+2m-3)(5lm+2m-6\right)}}+\frac{4(m-1)}{\left(5lm+2m-4\right)}\\ & + & \frac{2\left(4l+2m-4\right)\sqrt{5}}{\sqrt{(}5lm+2m-4)\left(5lm+2m-5\right)}\\ & + & \frac{2\left(4l+2m-4\right)\sqrt{6}}{\sqrt{\left(5lm+2m-4)(5lm+2m-6\right)}}\\ & + & \frac{5(18lm-7l-4m-5)}{\left(5lm+2m-5\right)}+\frac{\left(24lm-32l-14m+24\right)\sqrt{30}}{\sqrt{\left(5lm+2m-5)(5lm+2m-6\right)}}. \end{array}$$

16. Let $BN_{\left(l,m\right)}$ be Borophene *β*_12_-sheet. Then the F-temperature index is $\frac{8(m+2)}{{\left(5lm+2m-3\right)}^{2}}+\frac{\left(4l+2m-4\right)\left(128+500l^{2}m^{2}+80m^{2}+400m^{2}l\right)+\left(-480l-192\right)m}{{\left(5lm+2m-2\right)}^{2}{\left(5lm+2m-4\right)}^{2}}+\frac{18(m+2)}{{\left(5lm+2m-3\right)}^{2}}+\frac{\left(4l+2m-4\right)\left(288+625lm^{2}+100m^{2}+500lm^{2}\right)+\left(-840l-336\right)m}{{\left(5lm+2m-3\right)}^{2}{\left(5lm+2m-4\right)}^{2}}+\frac{4l\left(450+850l^{2}m^{2}+136m^{2}+680lm^{2}\right)+\left(-1200l-480\right)m}{{\left(5lm+2m-3\right)}^{2}{\left(5lm+2m-5\right)}^{2}}+\frac{\left(4l+2m\right)(648+1125l^{2}m^{2}+180m^{2}+900lm^{2}+\left(-1620l-648m\right)}{{\left(5lm+2m-3\right)}^{2}{\left(5lm+2m-6\right)}^{2}}+\frac{32(m-1)}{{\left(5lm+2m-4\right)}^{2}}+\frac{\left(4l+2m-4\right)\left(800+1025l^{2}m^{2}+164m^{2}+820lm^{2}\right)+\left(-1800l-720\right)m}{{\left(5lm+2m-4\right)}^{2}{\left(5lm+2m-5\right)}^{2}}+\frac{\left(4l+2m-6\right)\left(1152+1300l^{2}m^{2}+208m^{2}+1040lm^{2}\right)+\left(-2400l-960\right)m}{{\left(5lm+2m-4\right)}^{2}{\left(5lm+2m-6\right)}^{2}}+\frac{50(18lm-7l-4m-5)}{{\left(5lm+2m-5\right)}^{2}}+\frac{\left(24lm-32l-14m+24\right)\left(1800+1525l^{2}m^{2}+244m^{2}+1220lm^{2}\right)+\left(-3300l-1320\right)m}{{\left(5lm+2m-5\right)}^{2}{)\left(5lm+2m-6\right)}^{2}}$.

From Tables 9–12 along with Eq (9), we obtain

$$\begin{array}{ccc} FT(BN_{\left(l,m\right)}) & = & \left(m+2\right)[\{\frac{2}{(5lm+2m)-2}{\}}^{2}+\{\frac{2}{(5lm+2m)-2}{\}}^{2}]\\ & + & \left(4l+2m-4\right)[\{\frac{2}{(5lm+2m)-2}{\}}^{2}+\{\frac{4}{(5lm+2m)-4}{\}}^{2}]\\ & + & \left(m+2\right)[\{\frac{3}{(5lm+2m)-3}{\}}^{2}+\{\frac{3}{(5lm+2m)-3}{\}}^{2}]\\ & + & \left(4l+2m-4\right)[\{\frac{3}{(5lm+2m)-3}{\}}^{2}+\{\frac{4}{(5lm+2m)-4}{\}}^{2}]\\ & + & \left(4l\right)[\{\frac{3}{(5lm+2m)-3}{\}}^{2}+\{\frac{5}{(5lm+2m)-5}{\}}^{2}]\\ & + & \left(4l+2m\right)[\{\frac{3}{(5lm+2m)-3}{\}}^{2}+\{\frac{6}{(5lm+2m)-6}{\}}^{2}]\\ & + & \left(m-1\right)[\{\frac{4}{(5lm+2m)-4}{\}}^{2}+\{\frac{4}{(5lm+2m)-4}{\}}^{2}]\\ & + & \left(4l+2m-4\right)[\{\frac{4}{(5lm+2m)-4}{\}}^{2}+\{\frac{5}{(5lm+2m)-5}{\}}^{2}]\\ & + & \left(4l+2m-4\right)[\{\frac{4}{(5lm+2m)-4}{\}}^{2}+\{\frac{6}{(5lm+2m)-6}{\}}^{2}]\\ & + & \left(18lm-7l-4m-5\right)[\{\frac{5}{(5lm+2m)-5}{\}}^{2}+\{\frac{5}{(5lm+2m)-5}{\}}^{2}]\\ & + & \left(24lm-32l-14m+24\right)[\{\frac{5}{(5lm+2m)-5}{\}}^{2}+\{\frac{6}{(5lm+2m)-6}{\}}^{2}] \end{array}$$

After simplification, we get

$$\begin{array}{ccc} FT(BN_{\left(l,m\right)}) & = & \frac{8(m+2)}{{\left(5lm+2m-3\right)}^{2}}\\ & + & \frac{\left(4l+2m-4\right)\left(128+500l^{2}m^{2}+80m^{2}+400m^{2}l\right)+\left(-480l-192\right)m}{{\left(5lm+2m-2\right)}^{2}{\left(5lm+2m-4\right)}^{2}}\\ & + & \frac{18(m+2)}{{\left(5lm+2m-3\right)}^{2}}\\ & + & \frac{\left(4l+2m-4\right)\left(288+625lm^{2}+100m^{2}+500lm^{2}\right)+\left(-840l-336\right)m}{{\left(5lm+2m-3\right)}^{2}{\left(5lm+2m-4\right)}^{2}}\\ & + & \frac{4l\left(450+850l^{2}m^{2}+136m^{2}+680lm^{2}\right)+\left(-1200l-480\right)m}{{\left(5lm+2m-3\right)}^{2}{\left(5lm+2m-5\right)}^{2}}\\ & + & \frac{\left(4l+2m\right)(648+1125l^{2}m^{2}+180m^{2}+900lm^{2}+\left(-1620l-648m\right)}{{\left(5lm+2m-3\right)}^{2}{\left(5lm+2m-6\right)}^{2}}\\ & + & \frac{32(m-1)}{{\left(5lm+2m-4\right)}^{2}}\\ & + & \frac{\left(4l+2m-4\right)\left(800+1025l^{2}m^{2}+164m^{2}+820lm^{2}\right)+\left(-1800l-720\right)m}{{\left(5lm+2m-4\right)}^{2}{\left(5lm+2m-5\right)}^{2}}\\ & + & \frac{\left(4l+2m-6\right)(1152+1300l^{2}m^{2}+208m^{2}+1040lm^{2}\left(-2400l-960\right)m}{{\left(5lm+2m-4\right)}^{2}{\left(5lm+2m-6\right)}^{2}}\\ & + & \frac{50(18lm-7l-4m-5)}{{\left(5lm+2m-5\right)}^{2}}\\ & + & \frac{\left(24lm-32l-14m+24\right)\left(1800+1525l^{2}m^{2}+244m^{2}+1220lm^{2}\right)+\left(-3300l-1320\right)m}{{\left(5lm+2m-5\right)}^{2}{\left(5lm+2m-6\right)}^{2}}. \end{array}$$

### 3.2 Comparative analysis of temperature indices for $BN_{\left(l,m\right)}$

**Fig 4:**
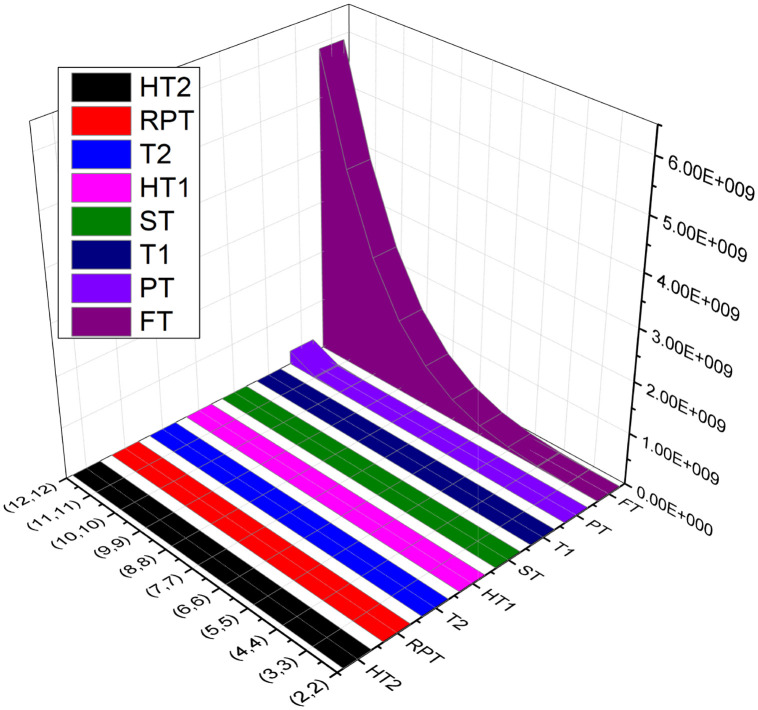
Graphical comparison of temperature indices of $BN_{\left(l,m\right)}$.

In this section, we perform a numerical and graphical comparison of temperature indices for *n* = 2, 3, 4, …, 12 of a Borophene *β*_12_-sheet $BN_{\left(l,m\right)}$. As we increase the value of *l* or *m*, the value of temperatures indices *T*_1_, *T*_1_, *T*_2_, *HT*_1_, *RPT* and *FT* decreased gradually while the numerical values of the temperature indices *HT*_2_, *ST* and *PT* increased gradually. These changes are also represented in Table 13 and Fig 4.

**Table 13:** Numerical values of temperature indices of $BN_{\left(l,m\right)}$ for *l* = *m*.

*p*	*T*_1_	*T*_2_	*HT*_1_	*HT*_2_	*ST*	*PT*	*RPT*	*FT*
(2,2)	678.43	88.562	578.39	0.50888	197.75	611.30	34.992	7.93×10^5^
(3,3)	740.5	97.421	735.08	0.043277	717.52	3263.4	35.802	5.99×10^6^
(4,4)	3329.4	104.91	834.26	0.0081426	1753.4	10453	36.968	2.51×10^7^
(5,5)	5446.0	110.53	903.44	0.0022568	3479	25607	37.931	7.63×10^7^
(6,6)	8090.3	114.80	954.56	0.0007918	6068.1	53117	38.695	1.89×10^8^
(7,7)	11263.0	118.13	993.89	0.000326	9693.9	98321	39.306	4.08×10^8^
(8,8)	14963.0	120.79	1025.1	0.000151	14530	1.6752×10^5^	39.799	7.93×10^8^
(9,9)	19191.0	122.96	1050.5	0.0000764	20749	2.6799×10^5^	40.206	1.42×10^9^
(10,10)	23947.0	124.76	1071.5	0.000041	28527	4.079×10^5^	40.547	2.41×10^9^
(11,11)	29231.0	126.28	1089.2	0.000023	38035	5.96×10^5^	40.836	3.88×10^9^
(12,12)	35042.0	127.57	1104.3	0.0000143	49448	8.44×10^5^	41.084	5.9×10^9^

## 4 Quantitative structure-activity property relationship model

Topological descriptors are numerical depictions of chemical compounds with information about the structure’s topology. Various descriptors have been learned and are often employed in QSPR to predict chemical characteristics and biological activities. These descriptors include physicochemical, constitutional, electrostatic, geometrical, and topological properties. The process begins with an appropriate molecular topological descriptor and concludes with an inference, hypothesis, or prediction about the molecule’s behaviour, characteristics, and attributes. High-quality experimental data must be available and accurate for this investigation to be successful. Determining a suitable topological descriptor for modeling in the analysis is the essential step in the QSPR/QSAR process. Since there is no consensus regarding the ideal molecular description, all conceivable descriptors are chosen. The capacity to predict a chemical compound’s properties, activities, and behaviour based on the molecular structure of related compounds whose properties, activities, and characteristics have already been assessed is a vital use of QSPR/QSAR models. Numerical values of the properties of borophene nanosheets are given in Table 14.

**Table 14:** Experimental values of the properties for the borophene nanosheet.

Descriptor	*β*_12_	*X* ^3^	2-pmmn
Shear modulus	68.5	60.5	94
Young’s modulus	179	198.5	398
Poisson’s ratio	0.176	0.116	-0.04

### 4.1 Linear regression model

Regression analysis aims to ascertain the significance and strength of the correlations via a numerical value provided by the topological index. Regression models come in a variety of forms, and which one you pick will depend on the kind of data you have and the issue you’re attempting to solve. The following are some popular varieties of regression models: exponential and logarithmic regression models, linear and polynomial regression models such as quadratic and cubic regression models, etc. We employ the linear regression models for a few properties of the borophene nanosheet obtained in this section. For the analysis, the model below is taken into account.

$$\begin{array}{c} Y=eK+f \end{array}$$

Where *Y* and *K* represent the property and structural descriptors. The analysis considers characteristics like Young’s modulus, Poisson’s ratio, and shear modulus. Regression analysis was done using the analytical features of Matlab. The regression model for the F-Temperature index is discovered to be the best fit for shear modulus based on the correlation coefficient (*R*), and the regression equation can be written as:

$$\begin{array}{c} Y=9.9221(FT)+48.103 \end{array}$$

The regression formula for shear modulus for the first hyper temperature index can be written as:

$$\begin{array}{c} Y=7.9653(HT_{1})+45.072 \end{array}$$

The regression model for the reciprocal product connectivity temperature index is discovered to be fit for Poisson’s ratio based on the correlation coefficient (*R*), and the regression equation can be written as:

$$\begin{array}{c} Y=1.2905(RPT)+14.129 \end{array}$$

The regression model for the second hyper temperature index is discovered to be fit for Young’s modulus based on the correlation coefficient (*R*), and the regression equation can be written as:

$$\begin{array}{c} Y=1770.4(HT_{2})+178.36 \end{array}$$

**Fig 5:**
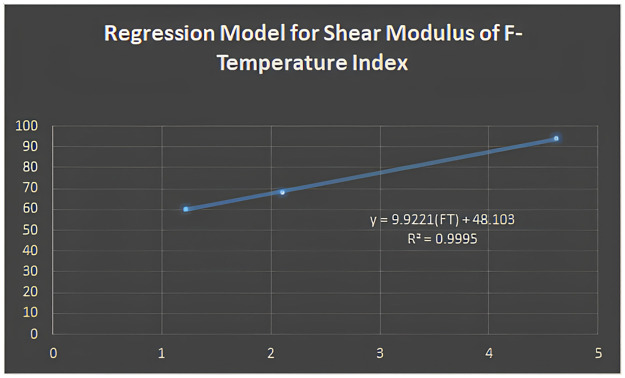
Regression model for shear modulus of F-Temperature index.

**Fig 6:**
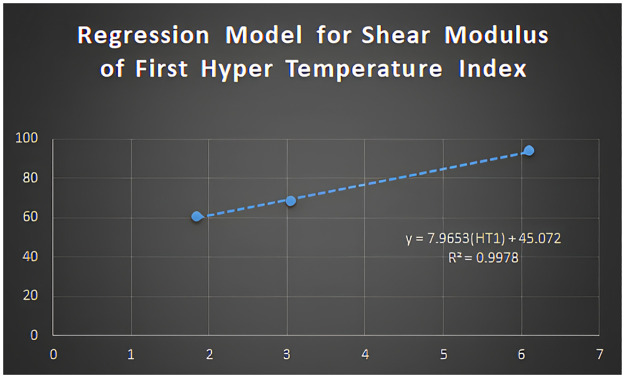
Regression model for shear modulus of first hyper-temperature index.

**Fig 7:**
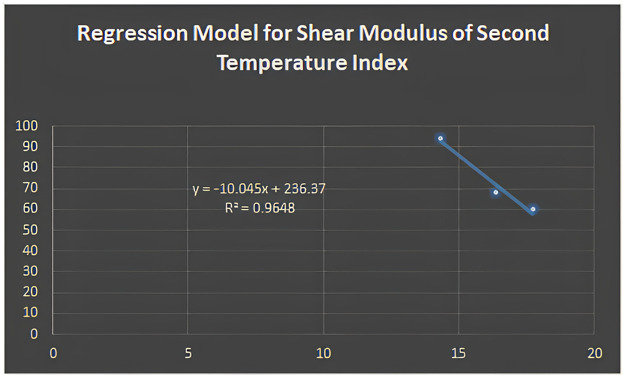
Regression model for shear modulus of second temperature index.

**Fig 8:**
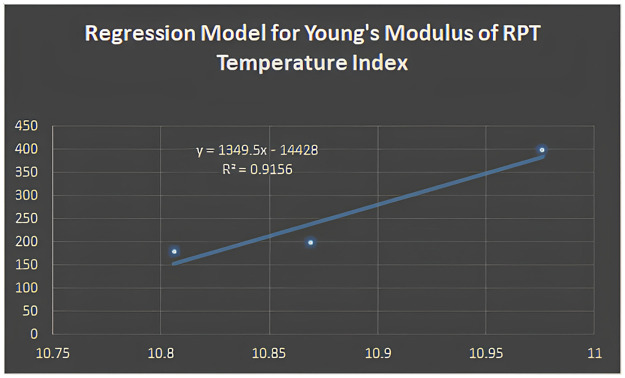
Regression model for young’s modulus of RPT temperature index.

**Fig 9:**
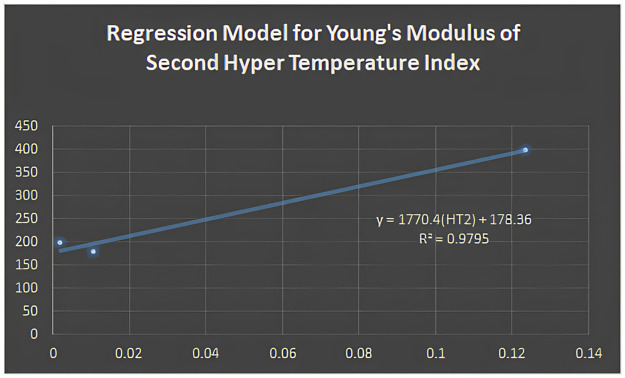
Regression model for young’s modulus of second hyper temperature index.

**Fig 10:**
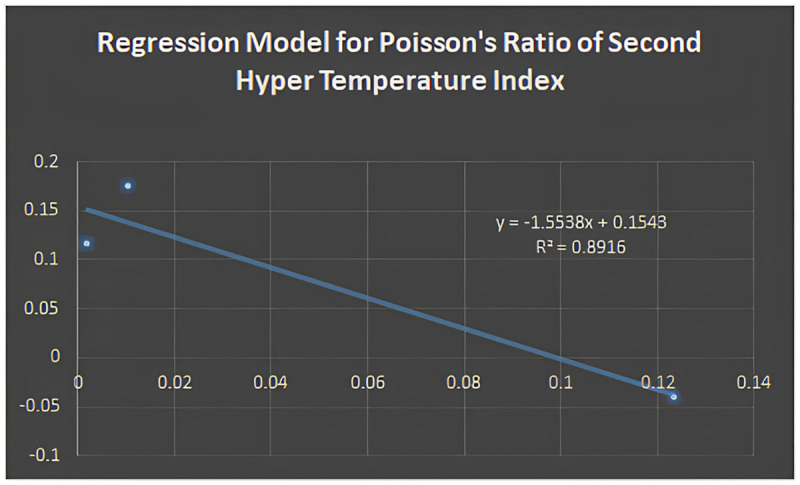
Regression model for poisson’s ratio of second hyper temperature index.

**Fig 11:**
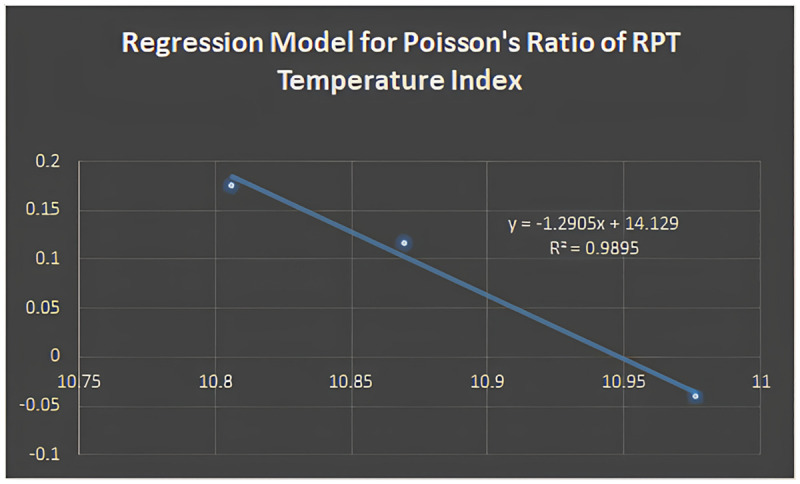
Regression model for poisson’s ratio of RPT temperature index.

Figs 5–11 depict regression models that show the relationship between attributes and their best-fitted descriptions. However, it can be concluded that the above-mentioned attributes of various boron variations can be anticipated using regression equations and calculated topological descriptors.

## 5 Conclusion

In this study, we computed degree-based temperature indices. We employed these indices to perform linear regression analysis to predict three physiochemical properties of borophene nanosheets: shear modulus, Young’s modulus, and Poisson’s ratio. We concluded that the linear regression analysis provides the following best-fit models with *R*^2^ value for shear modulus:

$$\begin{array}{c} y=9.9221\left(FT\right)+48.103;\;R^{2}=0.9995 \end{array}$$

$$\begin{array}{c} y=7.9653\left(HT_{1}\right)+45.072;\;R^{2}=0.9978 \end{array}$$

$$\begin{array}{c} y=-10.045\left(T_{2}\right)+236.37;\;R^{2}=0.9648 \end{array}$$

Young’s modulus:

$$\begin{array}{c} y=1770.4\left(HT_{2}\right)+178.36;\;R^{2}=0.9795 \end{array}$$

$$\begin{array}{c} y=1349.5\left(RPT\right)-14428;\;R^{2}=0.9156 \end{array}$$

$$\begin{array}{c} y=64.984\left(FT\right)+86.707;\;R^{2}=0.8933 \end{array}$$

and Poisson’s ratio:

$$\begin{array}{c} y=1.2905\left(RPT\right)+14.129;\;R^{2}=0.9895 \end{array}$$

$$\begin{array}{c} y=-1.5538\left(HT_{2}\right)+0.1543;\;R^{2}=0.8916 \end{array}$$

## Supporting information

S1 File (STY)
